# HCQ-SGuA: A Plant-Growth-Inspired Hybrid Saplings Growing Up Algorithm with Quasi-Random Initialization and Chaotic Operator Control for Continuous Optimization

**DOI:** 10.3390/biomimetics11090663

**Published:** 2026-09-16

**Authors:** Ebubekir Seyyarer

**Affiliations:** Department of Computer Engineering, Van Yüzüncü Yıl University, Van 65090, Türkiye; eseyyarer@yyu.edu.tr

**Keywords:** Saplings Growing Up Algorithm, chaotic maps, quasi-random initialization, hybrid metaheuristics, CEC2017, engineering design optimization, bio-inspired optimization

## Abstract

Metaheuristic optimizers often suffer from premature convergence, diversity loss, and stagnation in complex search landscapes. This study proposes HCQ-SGuA, a plant-growth-inspired framework that strengthens the Sowing phase through structured population initialization and regulates the biologically framed Mating, Branching, and Vaccinating mechanisms through chaotic operator control. An architecture-aware development–confirmation–holdout protocol was used to isolate initialization, chaotic control, their factorial interaction, and adaptive map selection on the 29 CEC2017 function classes available in OPFUNU 1.0.4 at *D* = 10 and *D* = 30. Faure initialization and Logistic control were the most consistent individual components, and high-budget confirmation selected HCQ-SGuA2[Faure + Logistic]. On the reserved holdout set, the frozen hybrid was competitive with its individual components and several established optimizers, but it did not significantly surpass the principal nonadaptive SGuA2 variants and remained inferior to DE and L-SHADE. The engineering experiments produced feasible and competitive best-run solutions, although mean performance and stability were weaker than those of the leading differential-evolution methods. The principal contribution is therefore a controlled biomimetic hybridization framework, showing when complementary interventions in Sowing and Growing Up cooperate and when added adaptive complexity is not beneficial.

## 1. Introduction

Optimization constitutes a fundamental problem-solving paradigm across a broad range of fields, including engineering, manufacturing, energy systems, finance, and artificial intelligence. From a mathematical perspective, continuous optimization seeks a decision vector within a feasible domain that minimizes or maximizes an objective function subject, where applicable, to equality and inequality constraints. Classical optimization theory provides rigorous frameworks for unconstrained and constrained problems, including gradient-based, trust-region, sequential quadratic programming, interior-point, and convex optimization methods [1,2]. Depending on the application, the objective may include maximizing efficiency or system performance, minimizing cost, computational complexity, or energy consumption, and reducing operational risk. However, when the search space is nonlinear, multimodal, constrained, discontinuous, or high-dimensional, obtaining satisfactory solutions may become computationally demanding, particularly when the structural assumptions required by conventional optimization methods are not satisfied. Consequently, heuristic and metaheuristic algorithms have become widely used for complex optimization problems in which analytical or deterministic approaches are difficult to apply [3,4,5,6,7,8,9].

Metaheuristic algorithms are high-level search methods designed to establish an effective balance between global exploration and local exploitation. Genetic Algorithms, Particle Swarm Optimization, Ant Colony Optimization, Differential Evolution, and many other population-based approaches belong to this class. The success of these algorithms depends not only on the mechanisms employed to generate and update candidate solutions but also on the spatial distribution of the initial population, the preservation of population diversity, and the quality of the stochastic decision mechanisms used throughout the search process [9,10]. Although many performance-improvement studies primarily focus on introducing new update equations or evolutionary operators, population initialization and controlled randomness can also have a substantial influence on convergence behavior, robustness, and final solution quality [10,11,12].

The Saplings Growing Up Algorithm (SGuA) is a nature-inspired population-based optimization method that models the sowing and growing processes of saplings through biologically motivated search mechanisms [13,14,15]. The algorithm consists of two principal phases. In the Sowing phase, an initial garden of candidate solutions is generated, whereas in the Growing Up phase, the population evolves through Mating, Branching, and Vaccinating operators. Unlike purely random initialization procedures, the original SGuA employs a partially structured sowing mechanism in which boundary information is used to generate some initial candidates and pseudo-random components are used to construct the remaining population. This mechanism introduces a certain degree of organization into the initial garden. Nevertheless, its ability to provide homogeneous, representative, and dimensionally scalable coverage of the search space can still be improved.

The initial population has a direct influence on the subsequent behavior of population-based optimization algorithms. A poorly distributed population may create overrepresented regions while leaving potentially promising areas insufficiently explored. This effect becomes increasingly important as dimensionality grows because a limited population must represent a rapidly expanding search space. Low-discrepancy sequences and other space-filling initialization strategies have therefore been investigated as alternatives to conventional pseudo-random initialization. Their objective is to reduce clustering and distribute initial candidates more uniformly throughout the feasible domain [16,17,18,19,20,21,22].

The sensitivity of SGuA to its Sowing phase has recently been examined more directly. Özgüner and Seyyarer [23] presented a comprehensive analysis of probability distribution-based initialization strategies for SGuA using classical and scalable benchmark functions, an overcurrent relay coordination problem, and a microgrid directional overcurrent relay coordination problem. Their results demonstrated that the selection of an initialization strategy can substantially affect convergence behavior, solution stability, and final optimization quality. The study also showed that the relative performance of different Sowing strategies may vary according to problem dimensionality, landscape characteristics, and constraint geometry. These findings provide important evidence that population initialization should be considered a fundamental algorithmic design factor rather than a secondary implementation detail.

However, improving the initial population alone cannot guarantee that diversity will be preserved throughout the entire optimization process. Even when the population initially covers the search domain effectively, repeated selection and information exchange may gradually concentrate candidate solutions around a limited number of regions. The pseudo-random control values used in the Mating, Branching, and Vaccinating mechanisms of SGuA therefore also play a decisive role in the algorithm’s iterative search dynamics. In complex and multimodal landscapes, insufficient initial coverage and progressive diversity loss may interact negatively, causing the search to concentrate prematurely around local optima while leaving other promising regions underexplored.

Chaotic maps provide a potential alternative to conventional pseudo-random control mechanisms. Although chaotic sequences are generated through deterministic equations, they may exhibit non-periodicity, ergodicity, sensitivity to initial conditions, and complex transition patterns. These properties have motivated their integration into different metaheuristic algorithms to improve population diversity, reduce stagnation, and strengthen local-optimum escape behavior [24,25,26,27,28,29,30]. Nevertheless, the effectiveness of a chaotic map is generally problem- and algorithm-dependent. A chaotic map that improves one evolutionary mechanism may not interact equally effectively with another operator or architectural structure. Therefore, chaotic integration should be evaluated empirically rather than assuming that a particular map is universally superior.

Another important consideration is that SGuA can be implemented through two related architectural forms. In the SGuA1 architecture, candidate populations produced by the Mating, Branching, and Vaccinating operators are generated from the current population and are subsequently evaluated within a common selection process. In contrast, the SGuA2 architecture employs a sequential structure in which evaluation and selection are performed after each evolutionary operator. These architectural differences may influence selection pressure, information propagation, diversity retention, and the interaction between the underlying algorithm and external initialization or chaotic-control mechanisms. Consequently, evaluating hybrid modifications on only one SGuA architecture may produce architecture-dependent conclusions and may conceal potentially effective combinations.

To address these issues, this study proposes HCQ-SGuA, a multi-stage Hybrid Chaotic–Quasi-Random Saplings Growing Up framework. Its biomimetic contribution is a coordinated intervention in two biologically analogous stages of plant development: structured, space-filling Sowing improves how the initial garden occupies the search habitat, while chaotic control modulates the Mating, Branching, and Vaccinating mechanisms during Growing-Up. The novelty lies not in claiming new low-discrepancy sequences or chaotic maps, but in testing their isolated and joint effects across both SGuA architectures before freezing the final hybrid.

Although quasi-random initialization and chaotic control have been investigated in other metaheuristics, the joint interaction among low-discrepancy Sowing, chaotic control of the three Growing Up operators, and the alternative SGuA1/SGuA2 information-flow architectures has not been systematically isolated. The study therefore distinguishes scientific contributions from validation safeguards. Its four principal contributions are as follows:

1. Architecture-aware factor isolation: SGuA1 and SGuA2 are retained while initialization and operator-control sources are varied under common evaluation rules.
2. Interaction-aware hybrid construction: initialization-only, chaos-only, and factorial hybrid configurations are compared so that component interaction is assessed rather than inferred from isolated rankings.
3. Static-versus-adaptive chaotic control: fixed-map hybrids are evaluated against an operator-specific adaptive map pool without assuming that adaptivity is inherently superior.

To evaluate these contributions without reusing selection information, the study employs a separate validation protocol comprising new-seed confirmation, configuration freezing, reserved-function holdout testing, engineering validation, and corrected nonparametric analysis. These procedures are treated as validation safeguards rather than algorithmic novelties.

The remainder of this article is organized as follows. Section 2 reviews related studies on population initialization, low-discrepancy sampling, chaotic search mechanisms, adaptive chaotic control, and SGuA-based optimization. Section 3 presents the theoretical background of the SGuA1 and SGuA2 architectures, the initialization strategies, and the chaotic maps considered in this study. Section 4 introduces the proposed multi-stage HCQ-SGuA framework, including its static and adaptive variants and the component-selection protocol. Section 5 describes the employed OPFUNU-based CEC2017 function set, the development–holdout partition, the engineering design problems, the parameter settings, the comparison algorithms, and the statistical evaluation procedure. Section 6 presents and discusses the architecture, initialization, chaotic-map, hybrid-screening, holdout, and engineering-design results. Finally, Section 7 summarizes the main findings, limitations, and potential directions for future research. For ease of reference, the principal symbols, abbreviations, and notation used throughout the manuscript are summarized in Table 1.

## 2. Related Works

Achieving an effective balance between global exploration and local exploitation remains one of the central challenges in metaheuristic optimization. Most population-based algorithms rely on pseudo-random number generators during population initialization, candidate generation, and operator-level decision processes. Although pseudo-random mechanisms provide diversity and stochastic behavior, they may also produce clustered samples, uneven spatial coverage, and insufficient representation of some regions of the search space. These effects become more pronounced in high-dimensional, multimodal, and constrained optimization problems, where inadequate initial coverage and progressive diversity loss may increase the risk of premature convergence and stagnation around local optima.

Two major research directions have been pursued to mitigate these limitations. The first focuses on improving the spatial quality of the initial population through space-filling sampling methods and low-discrepancy sequences such as Halton, Sobol, Faure, and Hammersley constructions. The second investigates the replacement or reinforcement of pseudo-random decision mechanisms with chaotic sequences, whose deterministic but non-periodic dynamics may contribute to diversity preservation and local-optimum escape. Although these approaches have been extensively investigated in different optimization algorithms, their effectiveness depends on the target problem, the underlying algorithmic architecture, and the stage at which they are integrated. This section reviews studies related to population initialization, chaotic search mechanisms, and the development of the Saplings Growing Up Algorithm.

### 2.1. Population Initialization and Low-Discrepancy Sampling

The distribution of candidate solutions at the beginning of a population-based optimization process can substantially affect convergence speed, search stability, and final solution quality. A well-distributed initial population increases the probability of representing different regions of the feasible domain, whereas a clustered population may direct the search toward a limited subset of the solution space before sufficient exploration has occurred [10,16,22].

Maaranen et al. [16] investigated quasi-random initial populations for genetic algorithms and demonstrated that low-discrepancy sequences such as Halton and Sobol can improve the spatial coverage of candidate solutions. Bangyal et al. [17] subsequently compared several low-discrepancy initialization approaches in population-based optimization algorithms and showed that the selected sequence can influence both convergence behavior and final optimization performance. Their findings also indicated that no single initialization method is consistently superior across all problem structures.

The use of low-discrepancy initialization has since been extended to different metaheuristic architectures. Kannan and Diwekar [18] incorporated quasi-random numbers into Particle Swarm Optimization to improve the distribution of particles and enhance convergence behavior. Zhang et al. [19] employed Sobol-based initialization in a Harris Hawks Optimization framework for high-dimensional data analysis. Yang et al. [20] integrated a Sobol sequence into an adaptive PSO–Snake Optimization structure for aerodynamic parameter identification, whereas Wang and Zhou [21] used Sobol initialization as part of a multi-strategy Whale Optimization Algorithm. These studies collectively indicate that space-filling initialization can reduce the unevenness associated with conventional pseudo-random sampling and may provide a more representative starting population.

However, the performance improvement produced by a low-discrepancy sequence is not independent of the underlying optimization algorithm. The interaction between the initial population and subsequent evolutionary operators may cause the same sequence to perform differently across algorithms, dimensions, and landscape types. Moreover, initialization affects only the beginning of the optimization process and does not directly guarantee that population diversity will be preserved during later iterations. As emphasized in the comprehensive review by Agushaka and Ezugwu [22], population initialization is an important design component, but its contribution should be considered together with the search and diversity-preservation mechanisms used throughout the optimization process.

The initialization methods considered in the literature also belong to different methodological classes. Latin Hypercube Sampling is a stratified space-filling method, whereas Halton, Sobol, Faure, Kronecker, Hammersley, and Van der Corput-based constructions are associated with low-discrepancy sampling. These strategies should therefore not be treated as mathematically identical, even though they share the common objective of obtaining a more representative distribution than conventional pseudo-random initialization. Their comparative evaluation under the same architecture, population size, seeds, and function-evaluation budget is necessary to isolate their actual effects.

### 2.2. Chaotic Maps in Metaheuristic Optimization

Even when the initial population provides satisfactory spatial coverage, population diversity may gradually decrease as selection and information-sharing mechanisms repeatedly favor similar candidate solutions. Chaotic maps have therefore been integrated into metaheuristic algorithms to modify the stochastic decisions used during iterative search. Chaotic sequences are generated through deterministic nonlinear equations but may exhibit ergodicity, non-periodicity, sensitivity to initial conditions, and complex transition behavior. These characteristics make them potential alternatives to conventional pseudo-random control values.

Alataş [24] developed chaotic variants of the Artificial Bee Colony algorithm and examined the contribution of different maps to global numerical optimization. Gandomi et al. [25] integrated chaotic dynamics into the Firefly Algorithm and reported improvements in search behavior for several benchmark problems. Saremi et al. [26] investigated chaotic Biogeography-Based Optimization and demonstrated that different chaotic maps may produce different exploration and exploitation characteristics.

Further chaotic adaptations have been proposed for more recent population-based algorithms. Zhao et al. [27] introduced an opposition-based chaotic Salp Swarm Algorithm for global optimization, while Kohli and Arora [28] incorporated chaotic dynamics into the Grey Wolf Optimizer for constrained optimization problems. Sayed et al. [29] employed a chaotic Crow Search Algorithm for feature selection, and Kaur and Arora [30] developed a chaotic Whale Optimization Algorithm. These studies demonstrate that chaotic control can affect convergence speed, population diversity, and the ability to escape local optima.

Nevertheless, the contribution of chaotic maps is strongly dependent on the map, operator, and problem structure. Logistic, Tent, Sine, Gauss, and Circle maps generate sequences with different distributions, transition patterns, and sensitivity characteristics. Therefore, the integration of a chaotic map should not be interpreted as automatically improving an optimization algorithm. A map that benefits one operator or problem class may produce unstable or excessively exploitative behavior in another. In addition, studies that compare chaotic variants without retaining a pseudo-random control configuration may not clearly isolate whether the observed improvement is caused by chaotic dynamics or by other implementation differences.

The cited chaotic metaheuristic studies commonly evaluate fixed-map variants, whereas operator-specific adaptive use remains less frequently examined [24,25,26,27,28,29,30]. This observation is limited to the reviewed literature and is not intended as a universal claim about all chaotic metaheuristics. It motivates evaluating fixed and adaptive map control within the same SGuA implementation.

### 2.3. Saplings Growing Up Algorithm and Its Variants

The Saplings Growing Up Algorithm was introduced by Karcı as a nature-inspired metaheuristic that models the sowing and growing processes of saplings [13,14,15]. The algorithm consists of a Sowing phase, in which an initial garden of candidate solutions is constructed, and a Growing Up phase governed by Mating, Branching, and Vaccinating operators. These operators simulate information exchange, structural development, and the transfer of beneficial characteristics among candidate solutions.

The theoretical basis and decision-making capability of SGuA were investigated in its foundational studies [13,14,15]. Subsequent research demonstrated that the algorithm could be adapted to different optimization domains. Demir et al. [31] applied SGuA to DNA motif discovery, while Özgüner and Seyyarer [32] examined optimization algorithms in overcurrent relay coordination. Hark et al. [33] employed SGuA in a multi-document summarization framework designed to optimize coverage and diversity simultaneously. These applications demonstrate that the SGuA operators can be adapted to numerical, engineering, bioinformatics, and information-processing problems.

A recent study by Özgüner and Seyyarer [23] provided a more direct examination of the influence of the Sowing phase on SGuA performance. The study evaluated ten probability distribution- and space-filling-based initialization strategies using 20 benchmark functions, an overcurrent relay coordination problem, and a microgrid directional overcurrent relay coordination problem. The results demonstrated that initialization has a measurable but problem-dependent influence on convergence, stability, and solution quality. In the benchmark experiments, beta-distribution-based initialization and Latin Hypercube Sampling produced strong overall results, whereas different initialization mechanisms became preferable in the ORC and microgrid DORC applications. These findings show that the initialization strategy should be selected according to the dimensionality, landscape characteristics, and constraint structure of the target problem rather than being treated as a fixed preliminary procedure [23].

The study in [23] represents an important advancement in understanding the initialization sensitivity of SGuA. However, its principal focus was the modification of the Sowing phase through probability distribution-based and space-filling strategies. The pseudo-random control mechanisms employed during the Mating, Branching, and Vaccinating processes were not jointly redesigned through chaotic dynamics. Furthermore, the interactions among low-discrepancy initialization, chaotic operator control, and the alternative SGuA1 and SGuA2 execution architectures were not examined through a common factorial experimental framework.

The distinction between SGuA1 and SGuA2 is particularly relevant when external search mechanisms are integrated. In SGuA1, candidate populations generated by the evolutionary operators are evaluated within a common selection structure, whereas SGuA2 performs evaluation and selection sequentially after each operator. These differences may influence selection pressure, diversity retention, and the propagation of information. Consequently, an initialization sequence or chaotic map that performs effectively within one architecture may not produce the same behavior within the other.

### 2.4. Research Gap and Positioning of the Proposed Framework

The reviewed literature establishes that initialization and chaotic control can influence search behavior, but that their effects are problem-, map-, operator-, and architecture-dependent. The unresolved scientific question addressed here is whether low-discrepancy Sowing and chaotic control of Mating, Branching, and Vaccinating produce complementary effects across the two SGuA execution architectures.

Accordingly, architecture, initialization, chaotic control, and their factorial interaction are treated as distinct experimental factors. Confirmation with new seeds, configuration freezing, and holdout evaluation are methodological safeguards used to test the robustness of the selected combination; they are not presented as independent gaps in the literature.

## 3. Theoretical Background

This section defines the optimization model and the SGuA concepts required to understand the proposed method. Detailed pseudocodes of the original SGuA procedures, implementation equations for the initialization strategies and chaotic maps, and numerical validity diagnostics are provided in Supplementary Sections S1–S4.

General continuous optimization model: All benchmark and engineering problems considered in this study are formulated within the standard framework of continuous constrained optimization [1,2]. Let $x=x_{1},\;\ldots ,\;x_{D}$ be the *D*-dimensional decision vector, *l* and *u* its lower and upper bounds, f(x) the objective function, and $g_{r}(x)\leq 0$ the r-th inequality constraint. Equality constraints, when present, are represented through a tolerance-based pair of inequalities. The feasible set and optimization task are defined as follows: minimize f(x) subject to $l_{j}\;\leq \;x_{j}\;\leq \;u_{j}\;(j=1,\ldots ,D),\;g_{r}(x)\;\leq \;0\;(r=1,\ldots ,m)$.

For unconstrained CEC2017 functions, *m* = 0. For engineering problems, feasibility is evaluated through the aggregate constraint violation $\Phi (x)=\sum_{r=1}^{m}max\{0,g_{r}(x)\}$, and candidate solutions are ordered by the scalar selection score defined in Section 4.2.1. This notation is used consistently throughout the equations and algorithms.

### 3.1. Saplings Growing Up Algorithm (SGuA)

The Saplings Growing Up Algorithm is a nature-inspired population-based optimization method introduced by Karcı to solve complex numerical optimization problems by modeling the sowing and growing processes of saplings [13,14,15]. The algorithm represents a population of candidate solutions as a garden of saplings and organizes the optimization process into two principal phases: Sowing and Growing-Up.

The Sowing phase constructs the initial garden, whereas the Growing Up phase evolves the population through three biologically motivated mechanisms: Mating, Branching, and Vaccinating. These mechanisms are intended to combine information exchange, structural variation, and directed modification while preserving competitive candidate solutions through deterministic or elitist selection.

#### 3.1.1. Sowing Phase

The Sowing phase constructs a structured initial garden from the variable bounds and continues until N saplings have been generated. The symbols $i_{e}$ and $I_{e}$ in the original procedure denote, respectively, the active coordinate index and the corresponding index set used during interval partitioning. The complete original procedure is provided as Supplementary Algorithm S1.

#### 3.1.2. Growing Up Phase and Operators

The Growing Up phase enables the transmission and transformation of information from one generation of saplings to the next. Unlike purely stochastic selection mechanisms such as roulette-wheel selection, SGuA employs a partially deterministic strategy in which the current garden is combined with newly generated candidate saplings, and the candidates with the best fitness values are retained. The Growing Up phase consists of three principal operators: Mating, Branching, and Vaccinating.

Mating Process: paired saplings exchange selected coordinates according to a distance-dependent matching probability; the complete original operator is provided as Supplementary Algorithm S2.

Branching Process: selected coordinates are perturbed according to the branch-activation relation; the complete original operator is provided as Supplementary Algorithm S3.

Vaccinating Process: information is transferred between sufficiently dissimilar saplings to introduce structurally different candidates; the complete original operator is provided as Supplementary Algorithm S4.

#### 3.1.3. Architectural Variants of SGuA

Architectural Variants of SGuA (SGuA1 and SGuA2): Depending on the problem characteristics, the algorithm can be run using two different strategies:

SGuA1 Architecture: Mating, Branching, and Vaccinating independently generate candidate gardens from the same current garden, followed by one collective elitist-selection step. The complete procedure is provided as Supplementary Algorithm S5 [13,14,15].

SGuA2 Architecture: each operator is followed immediately by evaluation and elitist selection, and the retained intermediate garden becomes the input to the next operator. The complete procedure is provided as Supplementary Algorithm S6 [13,14,15].

#### 3.1.4. Limitations and Development Motivation of the Original SGuA

The proposed modifications target two distinct limitations of the original continuous SGuA: finite pseudo-random Sowing may leave parts of the domain underrepresented, and pseudo-random Growing Up control may not preserve diversity during prolonged search. Section 4 evaluates these interventions independently before combining them.

### 3.2. Population Initialization Strategies

The distribution of candidate solutions in the initial population can substantially influence the early exploration behavior, convergence stability, and final solution quality of population-based optimization algorithms. Conventional pseudo-random sampling generates independent values throughout the search domain; however, a finite number of samples may exhibit regional clustering and leave some areas insufficiently represented. This limitation becomes more pronounced as the dimensionality of the problem increases.

Space-filling and low-discrepancy initialization strategies aim to obtain a more representative distribution of candidate solutions within a bounded search space. Nevertheless, these methods do not belong to a single mathematical class. Latin Hypercube Sampling is a stratified sampling method, whereas Van der Corput, Halton, Faure, Sobol, Kronecker, and Hammersley constructions are associated with low-discrepancy or quasi-random sampling. Therefore, their performance should be evaluated empirically under identical experimental conditions rather than assuming that one strategy is universally superior [22,23,24,25,26,27,28,29,30,31,32,33,34,35,36].

**Figure 1:**
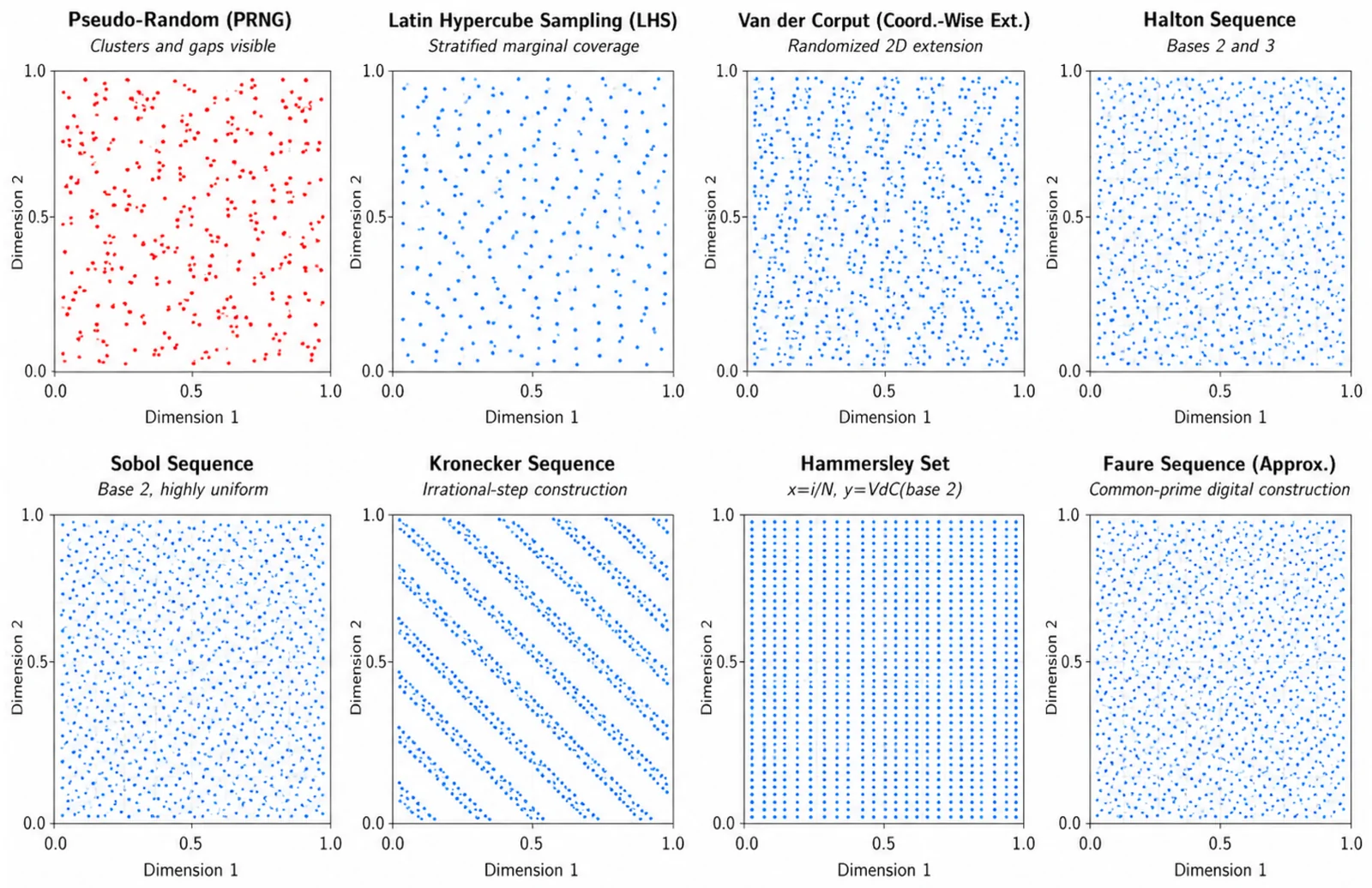
Two-dimensional distribution characteristics of the eight population initialization strategies considered in this study for *N* = 512: pseudo-random initialization, Latin Hypercube Sampling, randomized coordinate-wise Van der Corput, Halton, Faure, Sobol, Kronecker, and Hammersley.

The study evaluates PRNG, LHS, randomized coordinate-wise Van der Corput, Halton, Faure, Sobol, Kronecker, and Hammersley initialization. Figure 1 provides a two-dimensional visual comparison; Supplementary Table S1 and Supplementary Section S2 give the classifications, representative applications, and exact implementation equations [37,38,39,40,41,42,43,44,45,46,47,48,49,50,51,52,53,54,55]. No strategy is assumed to be superior a priori.

### 3.3. Chaotic Maps

Chaos theory describes the complex behavior of nonlinear deterministic systems that may exhibit non-periodicity, ergodicity, and strong sensitivity to initial conditions and control parameters. Although chaotic sequences are generated through deterministic mathematical equations, their temporal behavior may appear irregular and unpredictable. These characteristics have led to the use of chaotic maps in simulation, sampling, numerical analysis, secure communication, and metaheuristic optimization [56,57,58,59,60,61,62].

A one-dimensional chaotic map can generally be expressed as

(1) $$z_{k+1}=f_{C}\left(z_{k}\right),\;0<z_{k}<1,\;k=0,1,2,\ldots$$

In Equation (1), $z_{k}\;\in (0,1)$ denotes the normalized scalar state of chaotic controller C at controller step k, and $f_{C}(\cdot ):[0,1)\to [0,1)$ is the corresponding nonlinear state-update function. The function $f_{C}(\cdot )$ defines the nonlinear state update, whereas Equation (1) as a whole constitutes the recurrence relation that generates the controller-state sequence. The resulting sequence supplies normalized control values to the Mating, Branching, and Vaccinating operators. These values govern operator-level decisions involved in producing candidate solutions, which are subsequently evaluated according to the objective function f(x), the constraints $g_{r}(x)$, and the common selection score S(x). Thus, $z_{k}$ serves as an auxiliary controller state, whereas x denotes the optimization decision vector.

In metaheuristic optimization, chaotic sequences can be employed as alternatives to conventional pseudo-random control values. Their nonlinear transition patterns may affect information exchange, candidate modification, population diversity, and local-optimum escape behavior. Nevertheless, the contribution of a chaotic map depends on its mathematical structure, parameter settings, numerical implementation, and interaction with the underlying optimization operators. Therefore, no chaotic map is assumed to be universally superior.

**Figure 2:**
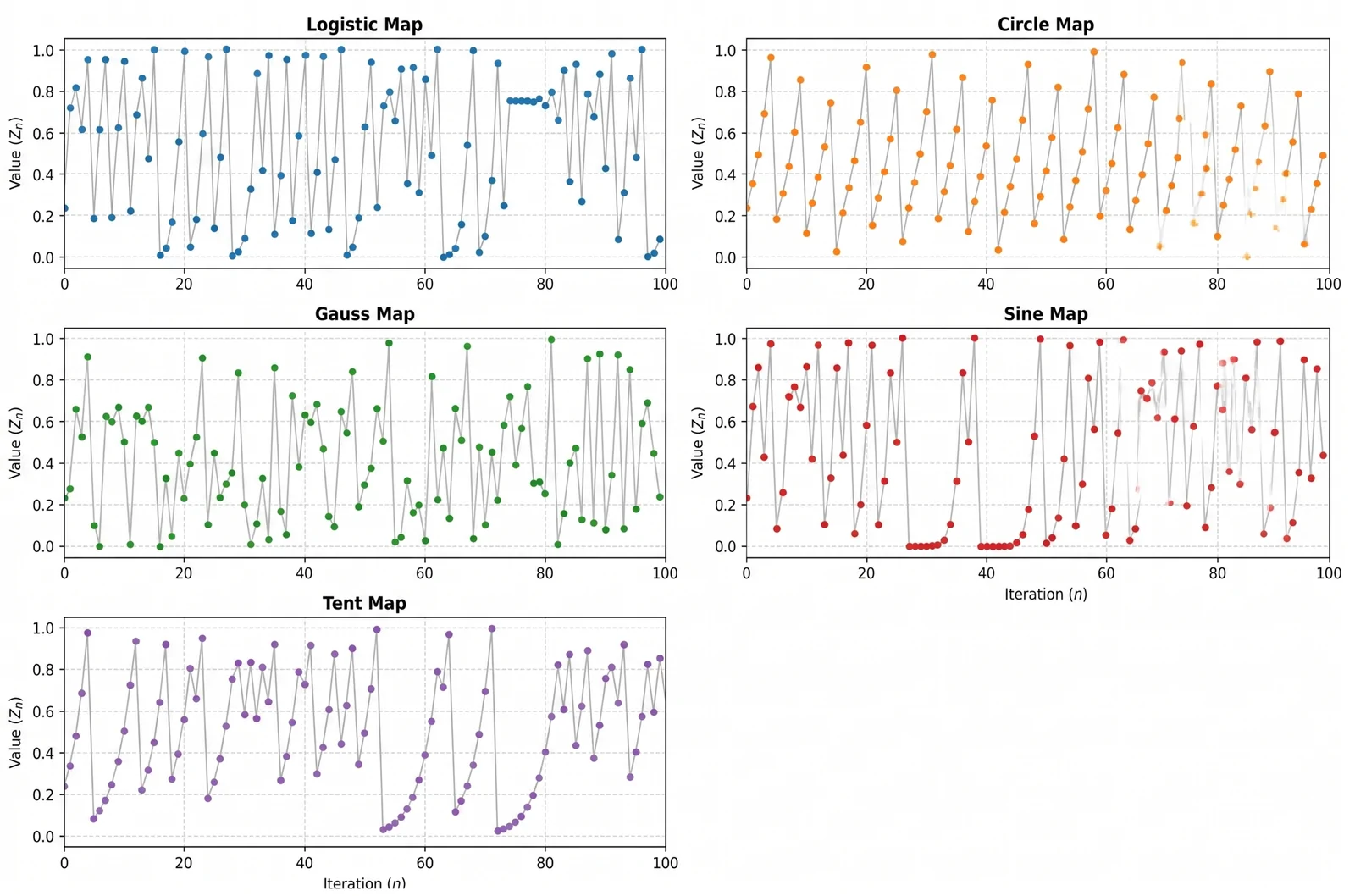
Time-series behavior of the five one-dimensional chaotic maps considered in this study under the same initial condition $\left(x_{0}=0.234\right)$. The plots illustrate the distinct transition patterns generated by the Logistic, Circle, Gauss, Sine, and Tent maps.

The study evaluates Logistic, Circle, Gauss, Sine, and Tent maps as operator-control sources. Figure 2 illustrates the time-series behavior of the five one-dimensional chaotic maps considered in this study under the same initial condition $\left(x_{0}=0.234\right)$. Their recurrence equations, parameterization, representative applications, and numerical validity diagnostics are provided in Supplementary Table S2 and Supplementary Sections S3 and S4 [63,64,65,66,67,68,69,70,71,72]. Each map is used through an independently initialized operator-specific stream after a 1000-step burn-in; PRNG control is retained as the reference.

The five chaotic maps considered in this study generate different transition patterns, marginal distributions, temporal dependencies, and sensitivities to initial conditions. These differences may influence their interactions with the Mating, Branching, and Vaccinating operators and may also produce architecture-dependent behavior in SGuA1 and SGuA2. Therefore, no chaotic map is selected theoretically in advance. Logistic, Circle, Gauss, Sine, and Tent maps are evaluated under identical population sizes, seeds, search bounds, and function-evaluation budgets, while the pseudo-random mechanism is retained as the reference configuration. The strongest chaotic candidates are determined through the Stage 2 experiments and are subsequently considered in the hybrid-screening stages.

## 4. Proposed Multi-Stage HCQ-SGuA Framework

This section presents the proposed multi-stage HCQ-SGuA framework developed to investigate and integrate alternative population initialization and iterative operator-control mechanisms within SGuA. Building on the theoretical foundations introduced in Section 3, the framework evaluates the effects of these mechanisms individually and jointly on both SGuA1 and SGuA2. The common computational core and the proposed method configurations are described in the following subsections.

### 4.1. Design Motivation and Framework Overview

The proposed framework is organized around two complementary modification axes corresponding to different stages of the SGuA search process. The first axis focuses on the Sowing phase and investigates space-filling and low-discrepancy initialization strategies to improve the distribution and representativeness of the initial population. The second axis focuses on the Growing Up phase and employs chaotic sequences as alternative control sources for the Mating, Branching, and Vaccinating operators.

The framework evaluates the effects of the SGuA architecture, population initialization strategy, and operator-control mechanism both independently and jointly. The initialization and chaotic-control mechanisms are first examined separately on the SGuA1 and SGuA2 architectures. The strongest component configurations identified through these analyses are then combined within static factorial and adaptive hybrid structures. This organization enables the individual contributions of the components and their interactions with the underlying SGuA architecture to be investigated under a common computational structure.

Accordingly, the proposed method family comprises four principal configurations:

1. QR-SGuA, in which the population initialization mechanism is modified using space-filling or low-discrepancy strategies, while the Growing Up operators retain pseudo-random control;
2. C-SGuA, in which the reference initialization mechanism is preserved, whereas the Mating, Branching, and Vaccinating operators are controlled using chaotic sequences;
3. Static HCQ-SGuA, in which an initialization strategy and a fixed chaotic map are jointly incorporated into the selected SGuA architecture;
4. Adaptive operator-weighted HCQ-SGuA, in which multiple chaotic maps are employed and their contributions are dynamically adjusted according to their observed effects during the search process.

These configurations provide a progressive methodological structure for examining the isolated effects of initialization and chaotic control, their fixed hybrid interaction, and the potential contribution of adaptive operator-level control. The detailed formulations of the four configurations are presented in the following subsections.

### 4.2. Common Continuous SGuA Computational Core

Because the benchmark and engineering-design problems considered in this study are defined in continuous search spaces, the Sowing, Mating, Branching, and Vaccinating principles of SGuA were operationalized through a common continuous-valued computational core [13,14,15]. This core was used consistently across all QR-SGuA, C-SGuA, static HCQ-SGuA, and adaptive HCQ-SGuA configurations. Consequently, the configurations differ only in their SGuA architecture, population initialization strategy, and operator-control source, while their boundary handling, candidate evaluation, operator formulations, selection rules, and function-evaluation accounting remain unchanged.

Let the current garden at iteration t be represented by

(2) $$G\left(t\right)=\left\{x_{1}\left(t\right),\;x_{2}\left(t\right),\;\ldots \;x_{N}\left(t\right)\right\},\;x_{i}\in R^{D}$$

where N is the population size and D is the problem dimension. Each decision variable is bounded by $l_{j}\leq x_{i,j}\leq u_{j},\;j=1,\;2,\;\ldots ,\;D$, where $l_{j}$ and $u_{j}$ denote the lower and upper bounds of the j decision variable.

The stochastic decisions required by the operators are obtained through a common controller interface. Depending on the evaluated configuration, this controller generates values using a pseudo-random number generator, a fixed chaotic map, or an adaptive pool of chaotic maps. Thus, the mathematical forms of the operators remain fixed, while only the source of their control values changes.

The SGuA1 and SGuA2 architectures use the same continuous operators but differ in their evaluation and selection schedules. In SGuA1, the three operators independently generate candidate groups from the same current garden, and all evaluated candidates are included in a single elitist selection step. In SGuA2, each operator is followed immediately by evaluation and elitist selection, and the selected intermediate garden becomes the input to the subsequent operator.

#### 4.2.1. Common Boundary-Handling and Selection Mechanisms

Candidate solutions generated by the continuous operators may move outside the permissible search range. To ensure that all evaluated solutions remain feasible with respect to the variable bounds, a modular reflection mechanism is applied coordinate-wise. Let

(3) $${∆}_{j}=u_{j}-l_{j}$$

denote the search interval of the j variable. For an out-of-bound candidate value $y_{j}$, the intermediate reflected coordinate is calculated as

(4) $$z_{j}=\left(y_{j}-l_{j}\right)mod\left(2{∆}_{j}\right)$$

The repaired value is then obtained as

(5) $${\hat{y}}_{j}=\left\{\begin{array}{c} l_{j}+z_{j},\;z_{j}\leq {∆}_{j}\\ l_{j}+2{∆}_{j}-z_{j},\;z_{j}>{∆}_{j} \end{array}\right.$$

This formulation can repair both small and large boundary violations without placing all infeasible candidates directly on the nearest boundary. For engineering-design problems requiring additional problem-specific repairs, the modular reflection is first applied, followed by the problem-specific repair rule and a final clipping operation to ensure $\hat{y}\in \left[l,u\right]$.

All candidate solutions are ranked according to a common scalar evaluation score. For unconstrained benchmark functions, this score is equal to the objective-function value. For constrained engineering problems, the total constraint violation is calculated as

(6) $$CV\left(x\right)=\sum_{r=1}^{M}\max\left(g_{r}\left(x\right),0\right)$$

where $g_{r}\left(x\right)\leq 0$ denotes the r−th inequality constraint, and (M) is the total number of inequality constraints. The scalar score used for selection is defined as

(7) $$S(x)=\left\{\begin{array}{c} f(x),\;CV\left(x\right)\leq 10^{-12}\\ 10^{12}+10^{10}CV\left(x\right)+\min(\left|f\left(x\right)\right|,10^{10}),\;CV\left(x\right)>10^{-12} \end{array}\right.$$

This common penalty formulation strongly prioritizes feasible solutions while preserving a consistent ordering among infeasible candidates according to their total constraint violations.

Elitist environmental selection is applied by combining the current garden with the relevant evaluated candidate group and retaining the N solutions with the lowest scores. Given a current garden G and an evaluated candidate set C, the next garden is obtained as

(8) $$G^{+}={Best}_{N}(G\;\cup \;C)$$

where ${Best}_{N}(\cdot )$ denotes ascending sorting according to S(x) and retention of the best N candidates.

For SGuA1, the selection pool includes the current garden and the candidate sets independently produced by all three operators:

(9) $$G(t+1)={Best}_{N}\left[G(t)\cup C_{M}(t)\cup C_{B}(t)\cup C_{V}(t)\right]$$

where $C_{M},\;C_{B}$, and $C_{V}$ denote the evaluated Mating, Branching, and Vaccinating candidate groups, respectively.

For SGuA2, selection is performed after each operator:

(10) $$G_{M}(t)={Best}_{N}\left[G(t)\cup C_{M}(t)\right],\;G_{B}(t)={Best}_{N}\left[G_{M}(t)\cup C_{B}(t)\right],\;\mathrm{and}\;G(t+1)={Best}_{N}\left[G_{B}(t)\cup C_{V}(t)\right]$$

Therefore, the two architectures differ in their information flow and selection pressure, while using identical operators and evaluation rules.

#### 4.2.2. Continuous Mating Operator

The continuous Mating operator exchanges decision-variable information between paired saplings. At the beginning of the operator, the indices of the current population are permuted using the active control source, and the saplings are grouped into non-overlapping pairs. If the population size is odd, the unpaired sapling remains in the current garden but does not produce a Mating candidate during that operator call.

For a pair of saplings $x_{a}$ and $x_{b}$, the normalized Mating probability is determined according to their Euclidean distance:

(11) $$P_{m}\left(x_{a},\;x_{b}\right)=max\left[0,\;1-\frac{{\left\|x_{a}-x_{b}\right\|}_{2}}{R}\right]$$

where

(12) $$R={\left\|u-l\right\|}_{2}$$

is the diagonal length of the bounded search space. Consequently, saplings located closer to one another have a higher probability of exchanging information.

Let $\xi_{m}\in [0,1)$ be a scalar value generated by the active operator controller. Mating is performed when $\xi_{m}\leq P_{m}\left(x_{a},\;x_{b}\right)$.

When the condition is satisfied, a component-wise binary mask is generated as

(13) $$m_{j}=\left\{\begin{array}{c} 1,\;\;\xi_{j}\leq p_{c}\\ 0,\;\;\mathrm{otherwise} \end{array}\right.\;\;\;\;\;\;\;\;\;\;p_{c}=0.50$$

where $\xi_{j}\in [0,1)$ is generated separately for each coordinate. If no coordinate is selected, one coordinate is chosen through the controller to guarantee information exchange.

Two complementary offspring are then produced:

(14) $$c_{1,j}=\left\{\begin{array}{c} x_{a,j},\;\;\;\;m_{j}=1\\ x_{b,j},\;\;\;\;m_{j}=0 \end{array}\right.$$

and

(15) $$c_{2,j}=\left\{\begin{array}{c} x_{b,j},\;\;\;\;m_{j}=1\\ x_{a,j},\;\;\;\;m_{j}=0 \end{array}\right.$$

Thus, the operator performs continuous component-level recombination without introducing new coordinate values outside those represented by the paired parents. The control source affects the pairing order, Mating decision, component mask, and forced-coordinate selection, while the mathematical structure of the operator remains identical across all configurations.

#### 4.2.3. Continuous Branching Operator

The continuous Branching operator introduces coordinate-level perturbations into each sapling. For every parent $x_{i}$, a binary activation mask is generated as

(16) $$b_{i,j}=\left\{\begin{array}{c} 1,\;\;\xi_{i,j}^{\left(1\right)}<p_{b}\\ 0,\;\;otherwise \end{array}\right.\;\;\;\;\;\;\;\;\;\;p_{b}=0.40$$

where $\xi_{i,j}^{\left(1\right)}\in \left[0,1\right)$ is supplied by the active controller.

For each coordinate, the direction of the perturbation is determined by

(17) $$d_{i,j}=\left\{\begin{array}{c} +1,\;\;\;\;\xi_{i,j}^{\left(2\right)}>0.5\\ -1,\;\;\;\;\xi_{i,j}^{\left(2\right)}\leq 0.5 \end{array}\right.$$

and a nonlinear scale variable is calculated as

(18) $$r_{i,j}=1+19\xi_{i,j}^{\left(3\right)}$$

where $\xi_{i,j}^{\left(2\right)}$ and $\xi_{i,j}^{\left(3\right)}$ are additional controller-generated values.

The coordinate-wise branching step is then defined as

(19) $$\delta_{i,j}=d_{i,j}{∆}_{j}\beta r_{i,j}^{-2},\;\;\;\;\;\;\;\;\;\;\beta =0.10$$

The resulting candidate is calculated as

(20) $$y_{i,j}=x_{i,j}+b_{i,j}\delta_{i,j}$$

The inverse-square term produces variable step sizes: smaller values of $r_{i,j}$ generate relatively larger movements, whereas larger values generate finer local perturbations. The factor ${∆}_{j}$ scales the movement according to the domain of each decision variable. After the perturbation, the boundary-repair mechanism in Equation (5) is applied.

One Branching candidate is formed for each current sapling. Therefore, the operator can simultaneously introduce local variations across the population while preserving unmodified coordinates through the activation mask.

#### 4.2.4. Continuous Vaccinating Operator

The continuous Vaccinating operator transfers directional information from relatively successful saplings to lower-quality members of the population. The current garden is first sorted in ascending order according to the common evaluation score and partitioned into two subsets:

(21) $$G^{good}=\left\{x_{\left(1\right)},\;\ldots ,\;x_{\left(\left\lfloor N/2\right\rfloor \right)}\right\}\;G^{poor}=G\backslash G^{good}$$

where $x_{\left(i\right)}$ denotes the i ranked solution.

For each poor sapling $x_{\left(a\right)}\in \;G^{poor}$, the donor is selected deterministically as the most distant member of the good subset:

(22) $$x_{d}=\arg\underset{x\in \;G^{\mathrm{good}}}{\max}\frac{{\left\|x-x_{a}\right\|}_{2}}{R}$$

Selecting the most distant successful donor introduces information from a structurally different region of the current population rather than repeatedly using the nearest elite solution.

The normalized coordinate-wise difference between the poor sapling and its donor is calculated as

(23) $$\delta_{j}=\frac{\left|x_{a,j}-x_{d,j}\right|}{\max\left({∆}_{j},10^{-15}\right)}$$

A candidate-specific threshold is obtained from the mean normalized difference:

(24) $$\bar{\delta }=\frac{1}{D}\sum_{j=1}^{D}\delta_{j}$$

Only the coordinates exhibiting an above-average difference are selected for vaccination:

(25) $$v_{j}=\left\{\begin{array}{c} 1,\;\;\;\;\;\;\delta_{j}>\bar{\delta }\\ 0,\;\;\;\;\;\;\mathrm{otherwise} \end{array}\right.$$

The vaccination coefficient is calculated as

(26) $$\gamma =\gamma_{max}\xi_{v},\;\;\;\;\;\;\gamma_{max}=0.60$$

where $\xi_{v\;}\in \left[0,1\right)$ is generated by the active operator controller.

The vaccinated candidate is then defined as

(27) $$y_{j}=\left\{\begin{array}{c} x_{a,j}+\gamma \delta_{j}(x_{d,j}-x_{a,j}),\;\;\;\;v_{j}=1\\ x_{a,j},\;\;\;\;v_{j}=0 \end{array}\right.$$

This formulation moves selected coordinates of the lower-quality sapling toward a successful but structurally distant donor. The normalized difference $\delta_{j}$ regulates the magnitude of the transfer, while γ controls its overall intensity. The repaired candidate is subsequently evaluated and enters the architecture-specific elitist selection process.

#### 4.2.5. Strict Function-Evaluation Accounting

A strict function-evaluation mechanism is used as the stopping criterion to ensure that all SGuA configurations operate under an identical computational budget. Let $FEs_{t}$ denote the number of objective-function evaluations completed before a new candidate group is evaluated. The remaining budget is calculated as

(28) $$B_{t}=MaxFEs-FEs_{t}$$

If an operator generates $n_{c}$ candidates, the number of candidates permitted for evaluation is restricted to

(29) $$n_{eval}=min(n_{c},\;B_{t})$$

Only these candidates are passed to the objective and constraint functions. After evaluation, the counter is updated according to

(30) $$FEs_{t+1}=FEs_{t}+n_{eval}$$

which guarantees

(31) $$FEs_{t}\leq MaxFEs$$

throughout every run.

The evaluations of the initial population are included in the same budget. Candidate generation, boundary repair, controller updates, ranking, and selection do not increment the evaluation counter because they do not invoke the objective function. In constrained problems, calculation of the objective and its associated constraints for a candidate is treated as one complete candidate evaluation under the common evaluator.

If the remaining budget is smaller than the number of candidates generated by an operator, only the number of candidates permitted by Equation (29) is evaluated. The unevaluated candidates are discarded, and no subsequent operator is executed after the budget is exhausted. This procedure also applies to an incomplete final SGuA generation.

The strict evaluator independently records the best solution encountered during the complete run, including candidates that may subsequently be excluded from the current population. Therefore, the final reported solution is the best evaluated solution rather than merely the highest-ranked member of the final garden. This function-evaluation protocol is applied consistently across the SGuA1 and SGuA2 architectures and all proposed method configurations.

The common computational core described in this subsection isolates the effects of population initialization, chaotic control, and SGuA architecture. The formulation of the initialization-enhanced QR-SGuA family is presented in the following subsection.

### 4.3. QR-SGuA: Initialization-Enhanced SGuA Family

The proposed configurations differ only in the SGuA execution architecture, population initialization strategy, and operator-control source, while the continuous operators and common computational mechanisms remain those defined in Section 4.2. To avoid repeating these common components, the methodological differences among the evaluated SGuA-derived configurations are summarized in Table 2.

Here, A∈{SGuA1,SGuA2}, Q denotes the evaluated population initialization strategy, and C denotes the chaotic control source.

QR-SGuA modifies only the population initialization stage. The initial garden is generated using one of the structured or low-discrepancy strategies introduced in Section 3.2, whereas the Mating, Branching, and Vaccinating operators retain pseudo-random control. Consequently, QR-SGuA isolates the contribution of population initialization without introducing chaotic control into the iterative search process.

All initialization strategies are evaluated independently on both SGuA1 and SGuA2 under identical population sizes, search bounds, seeds, and function-evaluation budgets. Their geometric characteristics are treated only as supporting diagnostics; the initialization candidates retained for subsequent hybrid construction are determined from the Stage 1 optimization results.

### 4.4. C-SGuA: Chaos-Controlled SGuA Family

C-SGuA modifies the operator-control mechanism of the common continuous SGuA core while preserving the reference PRNG initialization strategy. Specifically, the pseudo-random control values required by the Mating, Branching, and Vaccinating operators are replaced by values generated from one of the five chaotic maps introduced in Section 3.3. In this way, C-SGuA isolates the effect of chaotic operator control from changes in the spatial distribution of the initial population. The methodological distinction between C-SGuA and the other SGuA-derived configurations is summarized in Table 2.

For a selected chaotic control source C, the controller state evolves according to

(32) $$z_{k+1}=f_{C}\left(z_{k}\right),\;\;\;z_{k}\in [0,\;1)$$

where $z_{k}$ is the normalized scalar state of the selected operator-specific chaotic controller at controller step k, and $f_{C}(\cdot )$ is the nonlinear state-update function associated with the selected Logistic, Tent, Sine, Gauss, or Circle map. Equation (32) as a whole defines the recurrence relation used to generate the controller-state sequence. The initial controller state is sampled from U(0.05,0.95), and a burn-in period of 1000 controller steps is applied before the generated values are supplied as normalized control inputs to the Mating, Branching, and Vaccinating operators.

Because the five maps have different marginal distributions as well as different temporal dependence, the map comparison evaluates the complete controller-generating process rather than isolating a purely ‘chaotic’ effect.

During optimization, the generated chaotic values replace the pseudo-random values used for operator-level decisions. Depending on the operator, they control pair formation and activation in Mating, coordinate selection and perturbation characteristics in Branching, and transfer intensity in Vaccinating. The mathematical formulations, parameter settings, boundary-handling mechanism, elitist selection rules, and strict function-evaluation accounting remain identical to those defined in Section 4.2. Thus, the experimental difference between the reference configuration and C-SGuA arises only from the source of the operator-control values.

The same chaotic-map type is applied consistently to the three Growing Up operators, while operator-specific controller streams are initialized independently to avoid synchronized consumption of identical chaotic trajectories. Each map is evaluated separately on both SGuA1 and SGuA2 so that its interaction with the two architecture-specific information-flow and selection schedules can be examined without modifying the underlying continuous operators.

Before optimization, the chaotic-sequence validity diagnostics reported in Section 5 and Supplementary Section S4 were used to exclude collapsed, non-finite, or numerically unstable trajectories. These diagnostics are used only as numerical validity checks and not as criteria for selecting the strongest chaotic map. Final map selection is based on the Stage 2 optimization results obtained under identical population sizes, initial-population settings, search bounds, run seeds, benchmark functions, and function-evaluation budgets. The retained architecture–chaos configurations are subsequently considered in the static hybridization stage.

### 4.5. Static HCQ-SGuA Family

The static HCQ-SGuA family combines the initialization-oriented modification of QR-SGuA with the chaos-controlled operator mechanism of C-SGuA within a single configuration. In this structure, one population initialization strategy and one chaotic map are assigned to a selected SGuA architecture and remain fixed throughout the complete optimization run. Accordingly, the term static refers to the fixed initialization–chaos assignment and does not imply static search behavior. The methodological relationship of this configuration to the other SGuA-derived variants is summarized in Table 2.

For each architecture A, the set of static hybrid configurations is defined as

(33) $$H_{A}^{static}=\left\{HCQ-SGuA\left(A,\;Q,\;C\right):Q\in Q_{A}^{*},C\in C_{A}^{*}\right\},\;\;\;\;A\in \{SGuA1,\;SGuA2\}$$

Here, A identifies the SGuA execution architecture, $Q_{A}^{*}$ denotes the set of three architecture-specific initialization strategies retained from the QR-SGuA screening stage, and C denotes a selected chaotic map and $C_{A}^{*}$ = {Logistic, Tent, Sine, Gauss, and Circle} denotes the set of five validated chaotic maps. Therefore, the number of static configurations evaluated for each architecture is

(34) $$\left|H_{A}^{static}\right|=\left|Q_{A}^{*}\right|\;\times \left|C\right|=3\;\times \;5=15$$

Consequently, 15 static configurations are evaluated for each architecture, resulting in 30 architecture–initialization–chaos combinations across SGuA1 and SGuA2.

For a given static configuration, the selected strategy Q is used to construct the initial garden, whereas the selected chaotic map C supplies the control values required by the Mating, Branching, and Vaccinating operators throughout the run. As in C-SGuA, the three operators use independently initialized controller streams of the same chaotic-map type, including the common initial-state and burn-in settings described in Section 4.4. This prevents synchronized consumption of identical chaotic trajectories while preserving a fixed map family across the three operators. The continuous operator formulations, boundary handling, elitist selection, and strict function-evaluation accounting remain unchanged from the common computational core defined in Section 4.2.

The factorial construction is used to determine whether components that perform strongly in isolation remain effective when combined. In particular, a high-ranked initialization strategy under pseudo-random operator control does not necessarily form the strongest hybrid with every chaotic map, and the effectiveness of a chaotic controller may depend on the spatial characteristics of the initial population and the information-flow structure of the selected SGuA architecture. The factorial design therefore enables initialization–chaos–architecture interactions to be evaluated explicitly rather than selecting the final hybrid solely from the individual QR-SGuA and C-SGuA rankings.

### 4.6. Adaptive Operator-Weighted HCQ-SGuA

The Adaptive Operator-Weighted HCQ-SGuA (AOW-HCQ-SGuA) extends the static HCQ-SGuA family by allowing the chaotic control source to change dynamically during optimization. Unlike the static configuration, in which a single chaotic-map type remains fixed throughout the run, AOW-HCQ-SGuA enables the Mating, Branching, and Vaccinating operators to select independently among the five valid chaotic maps according to their observed search contributions. The population initialization strategy remains fixed and corresponds to the architecture-specific initialization candidate retained from the QR-SGuA stage. Thus, an adaptive configuration can be denoted as $AOW-HCQ-SGuA\left(A,Q_{A}^{*},C\right)$, where A∈{SGuA1,SGuA2} and C={Logistic,Tent,Sine,Gauss,Circle}.

Each Growing Up operator maintains an independent score for every chaotic map, with all scores initialized equally to zero. In addition, each operator–map pair has its own persistent chaotic-controller state. Therefore, even when the same map type is selected simultaneously by different operators, the corresponding sequences evolve independently and do not consume synchronized chaotic trajectories.

Operator-Specific Chaotic-Map Selection: At each operator call, one chaotic map is selected using a mixture of uniform exploration and score-guided exploitation. For map c and operator o, the selection probability is defined as

(35) $$P_{t}^{\left(o\right)}\left(c\right)=\frac{\varepsilon }{\left|C_{A}\right|}+(1-\varepsilon )\frac{\exp\left(s_{t}^{\left(o,c\right)}/\tau \right)}{\sum_{c^{\prime }\in C_{A}}\exp\left(s_{t}^{\left(o,c^{\prime }\right)}/\tau \right)}\;$$

where $s_{t}^{\left(o,c\right)}$ is the current utility score of map c for operator o, ε is the exploration probability, and τ is the softmax temperature. In the present implementation, ε=0.10 and τ=0.35. The uniform component preserves the possibility of selecting currently low-scoring maps, whereas the softmax component increasingly favors maps associated with stronger recent search outcomes.

Once a map is selected, its operator-specific controller stream supplies all scalar or vector control values required during that operator call. These values affect the same Mating, Branching, and Vaccinating decisions defined in Section 4.2; no changes are introduced into the underlying continuous operator formulations.

Architecture-Specific Reward Calculation: Because SGuA1 and SGuA2 employ different evaluation and selection schedules, the utility of a selected chaotic map is measured using architecture-specific rewards.

For SGuA1, all three operators generate offspring independently from the same parent garden before collective elitist selection. The reward is therefore based on both the best offspring improvement and the proportion of offspring outperforming the median parent score:

(36) $$R_{t}^{\left(o,c\right)}=I_{t}^{\left(o\right)}+0.01U_{t}^{\left(o\right)}$$

Here, $\Delta_{rel,o}=max\{0,(s_{parent}-s_{offspring})/max(|s_{parent}|,1)\}$ is the non-negative relative improvement produced by operator o, where $s_{parent}$ and $s_{offspring}$ are the best parent and offspring scores. qo is the fraction of that operator’s offspring whose scores are lower than the median parent score. Thus, the first term rewards direct best-score improvement and the second rewards a broadly competitive offspring set.

For SGuA2, each operator is immediately followed by evaluation and elitist selection. Its reward can therefore exploit the change between the garden entering the operator and the garden retained after selection:

(37) $$R_{t}^{\left(o,c\right)}=I_{t}^{\left(o\right)}+0.05D_{t}^{\left(o\right)}$$

where $I_{t}^{\left(o\right)}$ represents the non-negative normalized improvement in the best score between the pre- and post-selection gardens, and $D_{t}^{\left(o\right)}$ represents the positive change in population diversity. Diversity is evaluated in the normalized search space using the mean coordinate-wise standard deviation, so variables with different numerical ranges contribute comparably. Negative diversity changes are truncated to zero and are therefore not penalized directly.

Adaptive Score Update: After the reward is calculated, only the score associated with the map selected for the corresponding operator is updated. An exponential moving-average mechanism is employed:

(38) $$S_{t+1}^{\left(o,c\right)}=\left\{\begin{array}{c} \left(1-\alpha \right)S_{t}^{\left(o,c\right)}+{\alpha R}_{t}^{\left(o,c\right)},\;\;\;\;c=c_{t}^{\left(o\right)}\\ S_{t}^{\left(o,c\right)},\;\;\;\;c\neq c_{t}^{\left(o\right)} \end{array}\right.$$

where $c_{t}^{\left(o\right)}$ is the map selected for operator o and α=0.20 is the learning rate. Because the scores are maintained independently for Mating, Branching, and Vaccinating, the adaptive mechanism can learn different map preferences for different search operators rather than imposing a common map ranking across the complete algorithm.

Stagnation-Triggered Population Refresh: Adaptive map selection does not by itself guarantee recovery from prolonged population stagnation. AOW-HCQ-SGuA therefore includes a limited population-refresh mechanism. A stagnation counter is incremented whenever the global best score fails to improve beyond the numerical tolerance of 10^−15^ and is reset to zero after an improvement. Population refresh is activated when

(39) $$\eta \geq \eta_{max},\;\;\;\;\eta_{max}=12$$

where η denotes the current number of consecutive non-improving generations.

When the stagnation condition is satisfied, the worst-performing fraction of the current garden is replaced. The nominal refresh size is

(40) $$N_{refresh}=\max\left[1,\left\lceil \rho N\right\rceil \right],\;\;\rho =0.10$$

Replacement candidates are generated using the same fixed initialization strategy $Q_{A}^{*}$ assigned to the adaptive configuration, with new deterministic run-specific seeds for each refresh event. The new candidates replace the corresponding worst-ranked saplings, after which the garden is re-evaluated and re-ranked and the stagnation counter is reset. All replacement evaluations consume the remaining MaxFEs budget; when fewer evaluations remain than the nominal refresh size, the number of replacement candidates is reduced accordingly.

Role Within the Multi-Stage Framework: AOW-HCQ-SGuA is evaluated separately for SGuA1 and SGuA2 using the architecture-specific initialization strategy retained from Stage 1 and the complete five-map chaotic pool. The adaptive candidates participate in both the Stage 3A hybrid screening and the Stage 3B higher-budget confirmation stages under the same function-evaluation constraints as the corresponding static configurations. No performance advantage is assumed a priori; static and adaptive chaotic control are compared empirically under the common development–confirmation–holdout protocol.

In summary, AOW-HCQ-SGuA introduces three adaptive mechanisms beyond the static hybrid structure: operator-specific chaotic-map selection, reward-based map-score updating, and stagnation-triggered population refresh. All remaining components, including the continuous SGuA operators, boundary handling, elitist selection, initialization strategy within each run, and strict function-evaluation accounting, remain identical to the common computational core described in Section 4.2.

### 4.7. General Algorithmic Flow of the Proposed Framework

**Figure 3:**
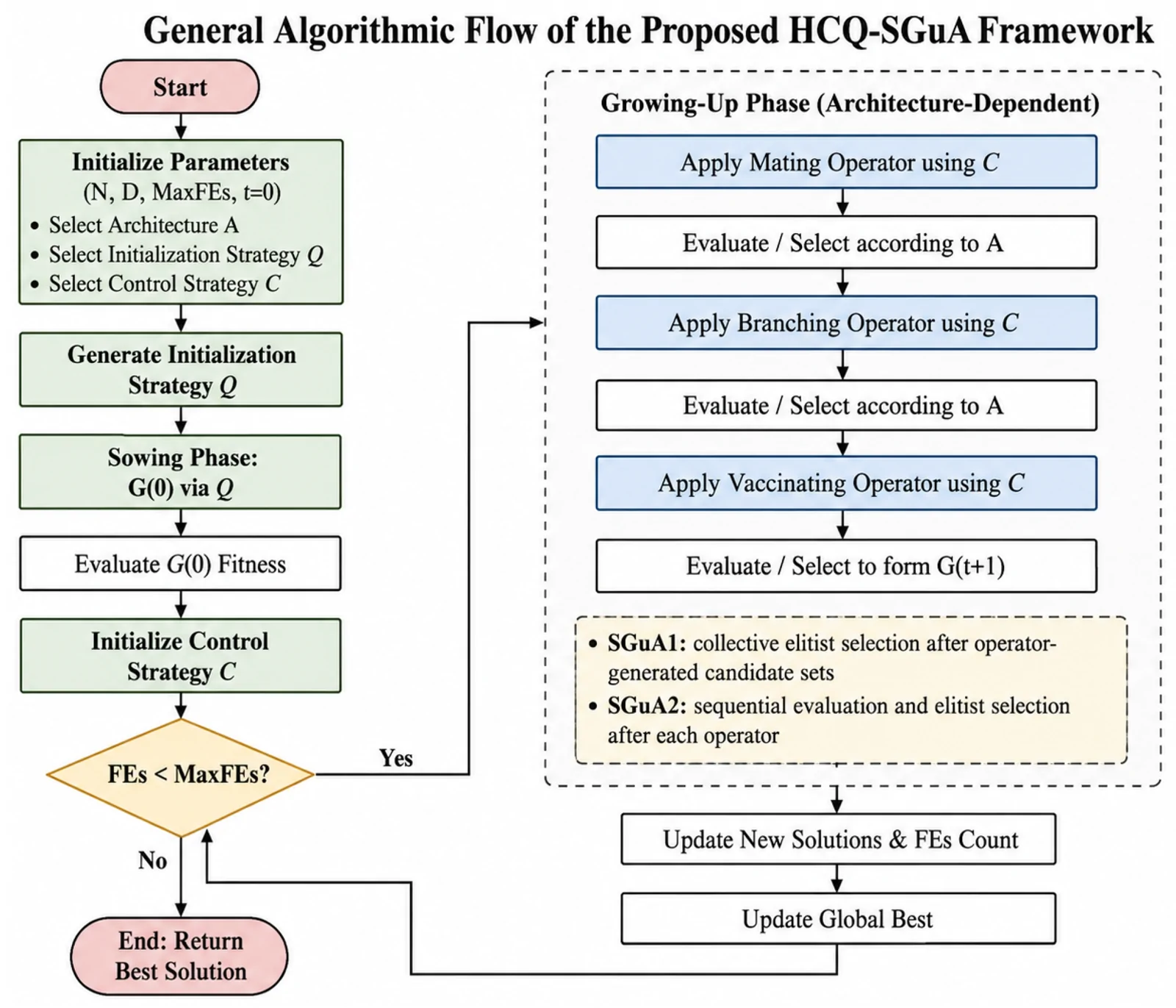
General algorithmic flow of the proposed HCQ-SGuA framework. The parameterized structure selects an SGuA architecture A, a population initialization strategy Q, and an operator-control strategy C. The Mating, Branching, and Vaccinating operators are executed according to the architecture-specific evaluation and elitist-selection schedule under a strict Maximum Function Evaluations (MaxFEs) budget.

Figure 3 summarizes the general computational flow of the proposed HCQ-SGuA framework. The framework is parameterized by the SGuA architecture A, population initialization strategy Q, and operator-control strategy C. Depending on these selections, the same computational structure represents QR-SGuA, C-SGuA, static HCQ-SGuA, or AOW-HCQ-SGuA.

At the beginning of each run, the initial garden is generated using Q, evaluated within the common MaxFEs budget, and the selected control mechanism C is initialized. The Mating, Branching, and Vaccinating operators are then executed according to the architecture-specific information flow defined in Section 4.2. SGuA1 applies collective elitist selection after generating the operator-specific candidate groups, whereas SGuA2 performs evaluation and selection sequentially after each operator. Static chaotic control retains a fixed map type with independent operator streams, while adaptive control follows the operator-specific selection and reward mechanism described in Section 4.6.

The process continues until the strict function-evaluation budget is exhausted. When the remaining budget is insufficient for a complete candidate group, only the permitted candidates are evaluated. The best solution encountered throughout the run is finally returned. Because the continuous operators, boundary handling, evaluation rules, and MaxFEs accounting are shared by all configurations, observed performance differences primarily reflect the selected architecture, initialization strategy, control mechanism, and their interactions.

### 4.8. Pseudocode of the Parameterized HCQ-SGuA Framework

Algorithm 1 summarizes the parameterized implementation of the proposed framework. The architecture A, initialization strategy Q, and control strategy C determine the evaluated configuration, while the continuous operators, boundary repair, elitist selection, and strict function-evaluation accounting remain common. Adaptive map-score updates and population refresh are activated only when C corresponds to AOW-HCQ-SGuA.
**Algorithm 1.** Pseudocode of the parameterized HCQ-SGuA frameworkInput:       Objective and constraint functions       Population size *N*       Problem dimension *D*       Lower and upper bounds *l* and *u*       Maximum function-evaluation budget *MaxFEs*       Architecture *A* ∈ {*SGuA1*, *SGuA2*}       Initialization strategy *Q*       Control strategy *C*       Adaptive parameters, when adaptive control is enabled Output:       Best evaluated solution *x_best_* and its objective value 1:   Set *t* ← 0 and *FEs* ← 0 2:   Generate the initial garden *G*(*t*) of size *N* using initialization strategy *Q* within [*l*, *u*] 3:   Evaluate *G*(*t*) without exceeding *MaxFEs* 4:   Update *FEs* and rank the evaluated saplings 5:   Set *x_best_* as the best solution encountered 6:   Initialize independent operator-control mechanisms for Mating, Branching, and Vaccinating:             a. independent PRNG streams, when *C* = *PRNG*;             b. independent streams of a fixed chaotic map, when *C* is static;             c. independent adaptive chaotic-map pools, when *C* is adaptive 7:   Set stagnation counter *η* ← 0 8:   While *FEs* < *MaxFEs* do 9:         Set *FEs_before_* ← *FEs* 10:       Store the current best score as before 11:       If *A* = *SGuA1* then 12:             Set evaluated candidate collection *E* ← ∅ 13:             For each operator o in {Mating, Branching, Vaccinating} do 14:                   If *FEs* ≥ *MaxFEs* then 15:                            Break 16:                     End If 17:                     Select or obtain the controller assigned to operator o according to control strategy *C* 18:                   Generate candidate set *Yo* by applying operator o to the current garden *G*(*t*) 19:                   Repair the candidate coordinates violating [*l*, *u*] 20:                   Evaluate only the candidates permitted by the remaining function-evaluation budget 21:                     Update *FEs* 22:                   If *C* is adaptive then 23:                         Calculate the operator-specific offspring reward 24:                            Update the score of the selected chaotic map 25:                     End If 26:                     Add the evaluated candidates to *E* 27:             End For 28:             Form *G*(*t* + 1) by retaining the best *N* saplings from *G*(*t*) ∪ *E* through collective elitist selection 29:      Else if *A* = *SGuA2* then 30:            Set *Gwork* ← *G*(*t*) 31:            For each operator o in {Mating, Branching, Vaccinating} do 32:                     If *FEs* ≥ *MaxFEs* then 33:                            Break 34:                     End If 35:                     Select or obtain the controller assigned to operator o according to control strategy *C* 36:                    Generate candidate set *Yo* by applying operator *o* to *Gwork* 37:                    Repair the candidate coordinates violating [*l*, *u*] 38:                    Evaluate only the candidates permitted by the remaining function-evaluation budget 39:                     Update *FEs* 40:                    Form the new *Gwork* by retaining the best *N* saplings from *Gwork* ∪ *Yo* 41:                    If *C* is adaptive then 42:                            Calculate the reward from the changes in solution quality and population diversity 43:                            Update the score of the selected chaotic map 44:                     End If 45:             End For 46:             Set *G*(*t* + 1) ← *Gwork* 47:      End If 48:       Update *x_best_* using all solutions evaluated during the iteration 49:       If the best score has improved beyond the numerical tolerance then 50:             Set *η* ← 0 51:      *Else* 52:             *Set*
*η* ← *η* + 1 53:       End If 54:       If *C* is adaptive and *η* reaches the stagnation limit and *FEs* < *MaxFEs* then 55:             Determine the number of saplings permitted for refresh                  according to the refresh ratio and remaining *FEs* 56:            Generate replacement candidates using initialization strategy *Q* 57:             Evaluate the replacement candidates and update *FEs* 58:             Replace the corresponding worst-ranked saplings in *G*(*t* + 1) 59:             Rank the updated garden and set *η* ← 0 60:       End If 61:       If *FEs* = *FEs _before_* then 62:            Break 63:       End If 64:       Set *t* ← *t* + 1 65:       Set *G*(*t*) ← *G*(*t* + 1) 66:  End While 67:  Return *x_best_* and *its* associated objective and constraint values

Algorithm 1 applies the strict evaluator defined in Section 4.2.5; therefore, incomplete candidate groups are truncated according to the remaining MaxFEs budget, and only objective/constraint evaluations increment the evaluation counter. The best solution is tracked over all evaluated candidates. Different selections of A, Q, and C produce the QR-SGuA, C-SGuA, static HCQ-SGuA, and AOW-HCQ-SGuA configurations summarized in Table 2.

### 4.9. Computational Complexity

Let N denote the population size, D the problem dimension, and $C_{eval}\left(D\right)$ the cost of evaluating one candidate solution. Population initialization requires approximately O(ND) operations. Within a Growing Up cycle, Mating and Branching require O(ND), whereas Vaccinating requires $O(N^{2}D)$ in the worst case because each lower-quality sapling may be compared with multiple candidate donors. Environmental selection additionally requires O(NlogN). Therefore, the worst-case computational cost of one complete Growing Up cycle is

(41) $$O(N^{2}D+NlogN+NC_{eval}(D))$$

Under the strict MaxFEs budget, the number of complete cycles is proportional to MaxFEs/N. Accordingly, the worst-case computational complexity of a complete optimization run can be expressed as

(42) $$O[MaxFEs(C_{eval}\left(D\right)+ND+\log N)]$$

Static chaotic control adds only O(1) overhead per generated control value. For AOW-HCQ-SGuA, map selection requires O(K) per operator call and the SGuA2 diversity calculation requires O(ND); because K=5 is fixed, these operations do not change the asymptotic complexity. Stagnation-triggered refresh also remains within the same MaxFEs budget. Both SGuA1 and SGuA2 require O(ND) asymptotic memory, since SGuA1 stores only a constant multiple of the population and SGuA2 processes candidate groups sequentially. Consequently, QR-SGuA, C-SGuA, static HCQ-SGuA, and AOW-HCQ-SGuA share the same asymptotic time and memory complexity, with practical runtime differences arising primarily from constant computational factors.

In the complexity expressions, N is population size, D is dimension, K=5 is the fixed map-pool size, $C_{eval}\left(D\right)$ is the cost of one objective/constraint evaluation, and MaxFEs is the evaluation budget. Equation (42) therefore includes the evaluation cost and the worst-case $O(N^{2}D)$ donor-search cost.

## 5. Experimental Setup

A multi-stage experimental protocol was designed to evaluate the individual and joint contributions of the SGuA architecture, population initialization strategy, and chaotic operator-control mechanism. The protocol separates component development from final performance assessment to reduce configuration-selection bias. Architecture diagnosis, initialization screening, chaotic-control screening, and hybrid construction are performed exclusively on a predefined development subset of the CEC2017 benchmark functions. The strongest static and adaptive candidates are subsequently re-evaluated using new seeds and a larger function-evaluation budget. Following this confirmation stage, the primary proposed configuration is frozen before evaluation on functions reserved for holdout evaluation.

All algorithms within the same experimental stage are evaluated using identical population sizes, search bounds, independent-run seeds, boundary-handling rules, and maximum function-evaluation budgets. The final holdout comparison includes the frozen HCQ-SGuA configurations and representative classical and advanced metaheuristic algorithms. The practical applicability of the proposed framework is additionally examined using three constrained engineering design problems. Performance is assessed through descriptive statistics, nonparametric significance tests, effect sizes, convergence behavior, runtime, and Win–Tie–Loss analysis.

Before optimization, initialization designs were characterized by centered discrepancy, minimum pairwise distance, dimensional rank, and coordinate correlation; chaotic streams were checked using range, finite-value proportion, lag-one autocorrelation, permutation entropy, and finite-time Lyapunov estimates. These diagnostics served only as implementation-validity and interpretation checks. They did not determine the winning configurations, which were selected from optimization results (Supplementary Section S4).

### 5.1. Computational Environment and Reproducibility

All experiments were implemented in Python 3.10.0 and executed in a Jupyter-based environment under Windows 11 Pro 64-bit. Numerical computation and result processing were performed using NumPy 1.26.0, SciPy 1.15.2, and pandas 2.2.3. The CEC2017 benchmark definitions [73] were accessed through OPFUNU 1.0.4 [74], while HBA, INFO, MGO, and GWO-WOA were executed through MEALPY 3.0.3. Matplotlib 3.10.1 was used for visualization. The experiments were executed on an AMD Ryzen 9 7950X3D 16-core processor with 128 GB RAM; the optimization computations were CPU-based and did not require GPU acceleration.

The CEC2017 benchmark functions were accessed through the OPFUNU implementation. Before execution, the experimental pipeline verified the availability of the required benchmark functions and checked that the development and holdout partitions were mutually exclusive and collectively covered the intended function set. The pipeline terminated when the required benchmark implementation or configuration was unavailable; no simplified or internally substituted benchmark function was used.

A fixed master seed of 20260731 was used to generate deterministic run-specific seeds. Within each experimental condition, competing algorithms were evaluated using identical run seeds, search bounds, population sizes, and function-evaluation budgets. Independent random or chaotic controller streams were maintained for population initialization and the Mating, Branching, and Vaccinating operators.

The raw results, configuration files, benchmark partitions, and retained candidate configurations were stored separately for each experimental stage. Periodic checkpointing allowed interrupted experiments to resume without repeating completed runs. The primary proposed configuration was recorded and frozen after the confirmation stage and before the holdout results were accessed. This procedure was adopted to support computational reproducibility and to prevent retrospective modification of the selected configuration based on holdout performance.

### 5.2. CEC2017 Benchmark Suite

The numerical experiments were conducted using the single-objective bound-constrained CEC2017 functions implemented in OPFUNU 1.0.4 [73,74]. The employed implementation provided 29 function classes indexed from F1 to F29, including shifted and rotated unimodal, multimodal, hybrid, and composition problems. Accordingly, all experiments and statistical comparisons reported in this study refer specifically to this implementation-defined F1–F29 set rather than to the official 29-function competition subset, in which F2 was excluded and F30 was retained.

For each candidate solution x, the reported optimization error was calculated by subtracting the known function bias from the value returned by OPFUNU:

(43) $$E\left(x\right)=f\left(x\right)-f_{bias}$$

Here, $f\left(x\right)$ denotes the raw objective value returned by the employed CEC2017 benchmark implementation, whereas $f_{bias}$ denotes the function-specific additive bias assigned to the corresponding benchmark function. This bias is equal to the objective value assigned to the known global optimum of that function. Accordingly, $E\left(x\right)=0$ represents attainment of the known global optimum. Because $f_{bias}$ is constant for all candidate solutions evaluated on a given function, its subtraction does not alter the ordering of candidate solutions or the optimization trajectory; it only expresses the reported objective value as an error relative to the known optimum. For each independent run, the reported final error was obtained by evaluating E(x) for the best solution found at the end of that run. The employed OPFUNU version did not provide an F30 class. Therefore, F30 was neither approximated nor replaced by a function from another implementation, thereby preserving implementation consistency across all experimental stages. Conversely, F2 was retained because it was available in the predefined OPFUNU function set used when the development–holdout partition was established. This implementation-specific choice should be considered when comparing the present results with studies following the official CEC2017 competition subset.

#### 5.2.1. Development and Holdout Partition

Before any algorithm results were examined, the OPFUNU F1–F29 implementation was partitioned using a category-stratified design: approximately 40% of the functions were assigned to development and the remainder to holdout while ensuring that both subsets contained unimodal, multimodal, hybrid, and composition problems. The development subset supported architecture/component decisions; the larger holdout subset was accessed only after configuration freezing. The assignment was based on function identity and category, not on HCQ-SGuA performance.

Both subsets therefore span the principal CEC2017 landscape categories, no function appears in both partitions, and the exact prespecified assignments are reported in revised Table 3 and the benchmark-partition manifest.

This separation prevents the holdout functions from influencing component selection, parameter decisions, or the construction of the final HCQ-SGuA configuration.

#### 5.2.2. Problem Dimensions and Search Budgets

The component-screening experiments were performed on the development functions at D=10 and D=30. Each configuration was evaluated over 15 independent runs using a maximum function-evaluation budget of

(44) MaxFEs=3000D

The shortlisted hybrid candidates were subsequently re-evaluated on the same development functions at D=10 and D=30 using 30 new independent runs and a higher confirmation budget of

(45) MaxFEs=10,000D

After configuration freezing, the final holdout evaluation was conducted at D=10 and D=30. Each frozen method was executed over 20 independent runs per function and dimension using.

The 10,000D confirmation budget was used only to test whether shortlisted candidates remained competitive over a longer search horizon under new seeds; it was not used to alter operators or tune parameters. The 5000D holdout budget deliberately returned to the prespecified deployment-oriented budget used during screening and tested whether the frozen choice transferred to a stricter regime. This two-budget design can still favor candidates with long-horizon behavior at the freezing step, so cross-budget sensitivity is acknowledged as a limitation rather than interpreted as evidence of universal superiority.

(46) MaxFEs=5000D

The initial-population evaluations were included in the corresponding function-evaluation budget. When the remaining budget was insufficient to evaluate a complete candidate group, only the number of candidates permitted by the available budget was evaluated. Thus, no algorithm exceeded the predefined MaxFEs limit.

### 5.3. Multi-Stage Experimental Protocol

The experimental protocol was organized into six consecutive stages to separate component analysis, hybrid construction, confirmation, and final testing. Architecture, initialization, and chaotic-control decisions were made using only the development functions. The primary configuration was frozen before the holdout results were accessed. The purpose and outputs of each stage are summarized in Table 4.

#### 5.3.1. Stage 0: Architecture Diagnostic

Stage 0 compared the reference SGuA1 and SGuA2 architectures using pseudo-random initialization and pseudo-random operator control. Both architectures were evaluated under identical development functions, dimensions, seeds, population sizes, and function-evaluation budgets. This stage was diagnostic only; neither architecture was eliminated, and both proceeded to the subsequent component-screening stages.

#### 5.3.2. Stage 1: Initialization-Strategy Screening

Stage 1 evaluated pseudo-random initialization, Latin Hypercube Sampling, randomized coordinate-wise Van der Corput, Halton, Faure, Sobol, Kronecker, and Hammersley strategies separately on SGuA1 and SGuA2. The Growing Up operators retained pseudo-random control so that performance differences could be attributed primarily to the initial population.

The configurations were ranked separately for each architecture. The three highest-ranked non-PRNG initialization strategies were retained for the static hybrid-screening stage, while the highest-ranked strategy was also used to construct the corresponding adaptive candidate.

#### 5.3.3. Stage 2: Chaotic-Control Screening

Stage 2 examined Logistic, Tent, Sine, Gauss, and Circle maps as alternative control sources for the Mating, Branching, and Vaccinating operators. A pseudo-random control configuration was retained as the reference, while population initialization was fixed to PRNG for all configurations.

The chaotic maps were ranked independently for SGuA1 and SGuA2. These rankings were used to identify the architecture-specific chaotic-control champion and to define the candidate maps included in the subsequent hybrid stages.

#### 5.3.4. Stage 3A: Static and Adaptive Hybrid Screening

Stage 3A combined the three highest-ranked non-PRNG initialization strategies from Stage 1 with all five chaotic maps from Stage 2. This produced 15 static HCQ-SGuA configurations for each architecture and 30 static configurations in total.

One adaptive operator-weighted candidate was also constructed for each architecture. The adaptive configuration used the corresponding architecture-specific initialization champion and an adaptive pool containing all five chaotic maps. Static and adaptive candidates were evaluated under the same development-stage settings. The highest-ranked static configurations were retained for confirmation.

#### 5.3.5. Stage 3B: High-Budget Confirmation and Configuration Freezing

Stage 3B re-evaluated the shortlisted candidates using new seeds, 30 independent runs, and the higher confirmation budget defined in Section 5.2.2. For each architecture, the confirmation set included:

- the three highest-ranked static configurations from Stage 3A;
- the direct combination of the independently identified Stage 1 and Stage 2 champions;
- the adaptive operator-weighted configuration.

Duplicate configurations were included only once. The confirmed candidates were ranked across the development functions, and the best overall candidate was designated as the primary proposed method. Its architecture, initialization strategy, and control mechanism were then recorded and frozen before Stage 4.

#### 5.3.6. Stage 4: Final Evaluation on Unseen Holdout Functions

Stage 4 evaluated the frozen proposed configurations on the holdout functions listed in Table 3. No architecture, parameter, initialization strategy, or chaotic-control decision was modified after the holdout results became available.

The final comparison included the primary proposed method, the retained SGuA ablation configurations, the two reference architectures, and the comparator algorithms described in Section 5.4. All methods were evaluated using identical holdout dimensions, run seeds, population sizes, and function-evaluation budgets.

The holdout evaluation was therefore restricted to the functions reserved from the OPFUNU-defined F1–F29 set. No post hoc replacement of F2 or addition of F30 was performed after the configuration-freezing stage.

#### 5.3.7. Stage 5: Constrained Engineering Design Problems

Stage 5 examined the practical applicability of the frozen HCQ-SGuA framework using three classical constrained engineering-design problems: welded beam design, pressure vessel design, and tension/compression spring design. The welded-beam and pressure-vessel problems contain four decision variables, whereas the spring-design problem contains three.

The exact objective functions, inequality constraints, auxiliary stress/deflection relations, variable bounds, and discrete-thickness repair used for all three engineering problems are given in Supplementary Section S5. The same mathematical models were used for every competing algorithm.

The welded-beam problem minimizes the fabrication cost of a welded beam through the weld thickness h, weld length l, beam height t, and beam thickness b. The formulation includes seven inequality constraints associated with shear stress, bending stress, geometric compatibility, fabrication cost, minimum weld thickness, end deflection, and buckling load. The search ranges were 0.1≤h, b≤2.0 and 0.1≤l, t≤10.0.

The pressure-vessel problem minimizes the material, forming, and welding cost of a cylindrical vessel through the shell thickness $T_{s}$, head thickness $T_{h}$, inner radius R, and cylindrical-section length L. Four constraints were imposed on the minimum shell thickness, minimum head thickness, required vessel volume, and maximum vessel length. The bounds were $0.0625\leq T_{s}$, $T_{h}\leq 6.1875$, 10≤R≤200, and 10≤L≤240. In accordance with the adopted manufacturing formulation, $T_{s}$ and $T_{h}$ were restricted to integer multiples of 0.0625 through a problem-specific repair operation.

The tension/compression spring problem minimizes spring weight by optimizing the wire diameter d, mean coil diameter $D_{c}$, and number of active coils $N_{c}$. Four inequality constraints were applied to account for deflection, shear-stress-related behavior, surge-related design requirements, and maximum outer diameter. The corresponding bounds were 0.05≤d≤2.0, $0.25\leq D_{c}\leq 1.3$, and $2\leq N_{c}\leq 15$.

The frozen HCQ-SGuA2 configuration was compared with the retained SGuA-derived variants and five external optimizers: PSO, DE, GWO, WOA, and L-SHADE. HBA, INFO, MGO, and GWO-WOA were not included in the engineering-design experiments. Each method was executed over 30 independent runs using a population size of N=100 and a maximum budget of 100,000 function evaluations.

All competing methods used identical objective and constraint formulations, search bounds, run-specific seeds, feasibility rules, and function-evaluation budgets. General bound violations were handled using the common reflection mechanism, followed, where applicable, by the corresponding problem-specific repair operation. Constraint violation was calculated using the common procedure defined in Section 4.2.1, and all objective and constraint evaluations were counted within the same strict MaxFEs budget.

### 5.4. Comparator Algorithms

The proposed framework was compared with five established population-based metaheuristic algorithms: Particle Swarm Optimization (PSO) [75], Differential Evolution (DE) [76], Grey Wolf Optimizer (GWO) [77], Whale Optimization Algorithm (WOA) [78], and Linear Population Size Reduction Success-History-Based Adaptive Differential Evolution (L-SHADE) [79]. These methods were selected to represent different search mechanisms, including velocity-based learning, differential mutation, leadership-guided search, encircling behavior, and adaptive parameter control. Four additional contemporary optimizers were included through their MEALPY implementations: the Honey Badger Algorithm (HBA) [80], INFO optimizer [81], Mountain Gazelle Optimizer (MGO) [82], and hybrid Grey Wolf–Whale Optimization Algorithm (GWO-WOA) [83]. HBA models the digging and honey-finding behavior of honey badgers; INFO employs weighted-mean updating, vector combination, and local search; MGO represents the social hierarchy and movement patterns of mountain gazelles; and GWO-WOA combines grey-wolf leadership with whale-inspired search behavior. The comparator set therefore included nine external optimizers representing evolutionary, swarm-based, mathematical, adaptive, and hybrid search mechanisms. Their main characteristics are summarized in Table 5.

The external comparator algorithms were evaluated together with the reference SGuA architectures and the retained QR-SGuA, C-SGuA, static HCQ-SGuA, and adaptive HCQ-SGuA configurations. The SGuA-derived variants were treated as internal component and ablation comparisons, whereas the nine algorithms listed in Table 5 constituted the external comparator set.

All methods were executed using the same benchmark functions, search bounds, run-specific seeds, and maximum function-evaluation budgets. The initial population size was set equally wherever supported by the algorithm structure. Function evaluations used for initialization were included in the common budget, and no method was permitted to exceed the prescribed MaxFEs value. The parameter settings of the proposed and comparator algorithms are presented in Section 5.5.

### 5.5. Algorithm Parameters and Fairness Controls

All algorithms were evaluated under identical problem definitions, search bounds, run-specific seeds, and maximum function-evaluation budgets. The initial population size was set to N=100 for all methods. For L-SHADE, this value represented the initial population size, while the algorithm’s native linear population-reduction mechanism was preserved. Evaluations performed during population initialization were included in the common MaxFEs budget.

The SGuA-derived configurations shared the same continuous Mating, Branching, and Vaccinating operators, boundary-repair mechanism, elitist-selection rules, and constraint-handling procedure. Therefore, the configurations differed only in the selected SGuA architecture, initialization strategy, and operator-control source. Comparator algorithms retained their defining update mechanisms and were implemented using the standard parameter settings associated with their original formulations [75,76,77,78,79]. The parameters used throughout the experiments are summarized in Table 6.

The common SGuA parameters were inherited from the reference continuous implementation and were not re-optimized on the holdout set. The adaptive parameters were fixed before Stage 3A: a stagnation patience of 12 avoids reacting to short non-improving intervals, while a 0.10 refresh fraction limits disruption to the worst-ranked tail of the garden; ε=0.10 preserves map exploration, τ=0.35 provides moderate score sensitivity, and the learning rate controls gradual rather than abrupt score replacement. These values are design settings rather than empirically optimal constants.

For paired comparisons, the same run-specific seed was assigned to every competing method under the same function, dimension, and run index. This procedure ensured reproducible replications and reduced variation attributable to different random realizations. No method was permitted to use additional objective-function evaluations, restart evaluations, or population-refresh evaluations beyond the specified budget.

The initial population size was equalized, but architecture-specific operations such as L-SHADE’s population reduction and the adaptive map-selection mechanism of AOW-HCQ-SGuA were retained because they constitute defining components of the corresponding algorithms. Runtime was measured separately and was not used as a stopping criterion.

### 5.6. Performance Measures and Statistical Analysis

For the CEC2017 functions, optimization performance was evaluated using the final error value defined in Section 5.2. Lower values indicate better performance, and a value of zero represents attainment of the known global optimum. For each method–function–dimension combination, the mean, standard deviation, median, interquartile range, best value, and worst value were calculated over the independent runs. Mean runtime and the number of consumed function evaluations were also recorded. For the constrained engineering problems, the final objective value, feasibility rate, and mean constraint violation were additionally reported. For the magnitude analysis, the relative objective-value difference was computed for each problem as $\Delta_{rel}=100(fcomp-fHCQ)/max(\left|fcomp\right|,\;\left|fHCQ\right|,10^{-12})$ and then averaged across the relevant problem set; positive values favor HCQ-SGuA2.

Let $e_{a,f,d,r}$ denote the final bias-corrected error obtained by algorithm a on function f at dimension d in independent run r, and let R denote the total number of independent runs conducted for the corresponding experimental condition. The mean error is calculated over all independent runs as

(47) $${\bar{e}}_{a,f,d}=(1/R)\sum_{r=1}^{R}e_{a,f,d,r}$$

Thus, for a fixed algorithm a, function f, and dimension d, the summation in Equation (47) includes the final errors obtained in all R independent runs.

For a fixed dimension d, algorithms are ranked separately on each function according to ${\bar{e}}_{a,f,d}$, with rank 1 assigned to the lowest mean error. Let F denote the set of evaluated functions and let F = |F| be the number of functions in that set. The dimension-specific average rank is calculated as

(48) $${\bar{r}}_{a,d}=(1/F)\sum_{f\in F}rank({\bar{e}}_{a,f,d})$$

Exact ties at the stored numerical precision receive average ranks. The cross-dimensional aggregate rank is the arithmetic mean of the dimension-specific average ranks obtained at D = 10 and D = 30:

(49) $${\bar{r}}_{a}^{agg}=\frac{{\bar{r}}_{a,10}+{\bar{r}}_{a,30}}{2}$$

For a primary algorithm A and comparator B, W–T–L counts the functions for which the mean error of A is lower than, equal to at the stored numerical precision, or higher than the mean error of B, respectively. A ‘significant win’ requires both a Holm-adjusted paired-test *p*-value below 0.05 and an effect-size direction favoring A; rank alone is not interpreted as statistical significance.

Statistical differences among multiple algorithms were examined using the Friedman test [84]. When the omnibus test indicated a significant difference, two-sided Wilcoxon signed-rank tests [85] were conducted between the primary HCQ-SGuA configuration and each competing method. The resulting *p*-values were adjusted using Holm’s sequential procedure [86] to control the family-wise error rate. Statistical significance was assessed at α = 0.05.

In addition to the comparisons across benchmark functions, seed-matched run results were analyzed separately for each function–dimension pair using the Wilcoxon signed-rank test. Holm adjustment was applied within each comparison family. The magnitude and direction of pairwise differences were quantified using the Vargha–Delaney $A_{12}$ effect-size measure [87]:

(50) $$A_{12}=P\left(X_{A}<X_{B}\right)+\frac{1}{2}P(X_{A}=X_{B})$$

where $X_{A}$ and $X_{B}$ denote the final errors obtained by the primary method and a comparator, respectively. Since all problems were formulated as minimization tasks, $A_{12}>0.5$ favors the primary method, $A_{12}<0.5$ favors the comparator, and $A_{12}=0.5$ indicates equivalent stochastic performance.

Convergence behavior was evaluated using the median best-so-far error across independent runs at common function-evaluation checkpoints. This representation enabled convergence comparisons under an identical computational budget without allowing differences in iteration structure to affect the evaluation.

## 6. Results and Discussion

### 6.1. Architecture Diagnostic Results

Stage 0 compared the SGuA1 and SGuA2 architectures using identical PRNG-based initialization and operator control. At D = 10, SGuA1 achieved a lower mean error on 8 of the 12 development functions and obtained an average rank of 1.333, compared with 1.667 for SGuA2. At D = 30, each architecture achieved the lower mean error on six functions, resulting in an equal average rank of 1.500. The architecture-level results are summarized in Table 7.

The paired Wilcoxon tests showed no statistically significant architectural difference on any development function at D=10. At D=30, SGuA2 performed significantly better on F10 (*p* = 0.0413) and F28 (*p* = 0.0151), whereas the remaining comparisons were not significant at α=0.05. Thus, the results do not indicate a universal statistical advantage for either architecture.

Based on the aggregate ranking across both dimensions, SGuA1-PRNG was designated as the architecture diagnostic winner. Nevertheless, the difference between the two architectures was limited, and SGuA2 showed clear advantages on several higher-dimensional problems. Therefore, Stage 0 was treated as a diagnostic comparison rather than an elimination stage, and both architectures were retained for the subsequent initialization, chaotic-control, and hybrid-screening experiments.

### 6.2. Initialization-Strategy Screening Results

Stage 1 compared seven structured initialization strategies with the PRNG reference separately for SGuA1 and SGuA2. The methods were ranked across the 12 development functions at D=10 and D=30, with lower average ranks indicating better performance. The leading results and the strategies retained for hybrid construction are summarized in Table 8.

For SGuA1, Faure achieved the best rank at D=10, whereas Sobol ranked first at D=30. Nevertheless, Faure obtained the lowest cross-dimensional average rank of 3.625, followed by Sobol and Latin hypercube sampling. These three strategies were therefore retained for the SGuA1 hybrid-screening stage.

A similar dimension-dependent pattern was observed for SGuA2. Sobol ranked first at D=10, while Faure achieved the best rank at D=30. Faure again produced the lowest overall average rank of 3.625, with Sobol and Hammersley forming the remaining two retained non-PRNG strategies.

The results show that no single initialization method was superior at every dimension and architecture. However, Faure provided the most consistent aggregate performance and was consequently selected as the initialization champion for both SGuA1 and SGuA2. Sobol remained a strong alternative, particularly at D=30 for SGuA1 and D=10 for SGuA2. These findings also indicate that structured initialization does not automatically improve every configuration; its contribution depends on the interaction between the sampling strategy, architecture, and problem dimension.

### 6.3. Chaotic-Control Screening Results

Stage 2 compared five chaotic maps with the PRNG-control reference while retaining PRNG initialization. The methods were ranked separately for each architecture across the 12 development functions at D=10 and D=30. The architecture-specific results are summarized in Table 9.

The numerical validity of the chaotic streams was additionally verified using the diagnostics reported in Supplementary Section S4, including permutation entropy [88] and finite-time Lyapunov estimates [89]; these measures were used only as validity checks and not as map-selection criteria.

For SGuA1, the Logistic map achieved the best rank at D=10. At D=30, Logistic, Sine, and PRNG control obtained the same average rank; however, Logistic produced the lowest cross-dimensional average rank of 2.208. Gauss and Sine followed with equal overall ranks of 2.500.

For SGuA2, Sine ranked first at D=10, whereas Logistic and PRNG control shared the leading rank at D=30. The performance of Sine deteriorated as dimensionality increased, while Logistic remained competitive under both settings and achieved the best overall rank of 2.167.

Tent and Circle consistently occupied the lowest positions for both architectures. These findings demonstrate that chaotic control does not necessarily improve search performance and that its effectiveness depends on the map dynamics, architecture, and dimensionality. Based on cross-dimensional consistency, Logistic was selected as the chaotic-control champion for both SGuA1 and SGuA2. Nevertheless, all five chaotic maps were retained in the static factorial hybrid screening conducted in Stage 3A.

### 6.4. Static and Adaptive Hybrid Screening

**Figure 4:**
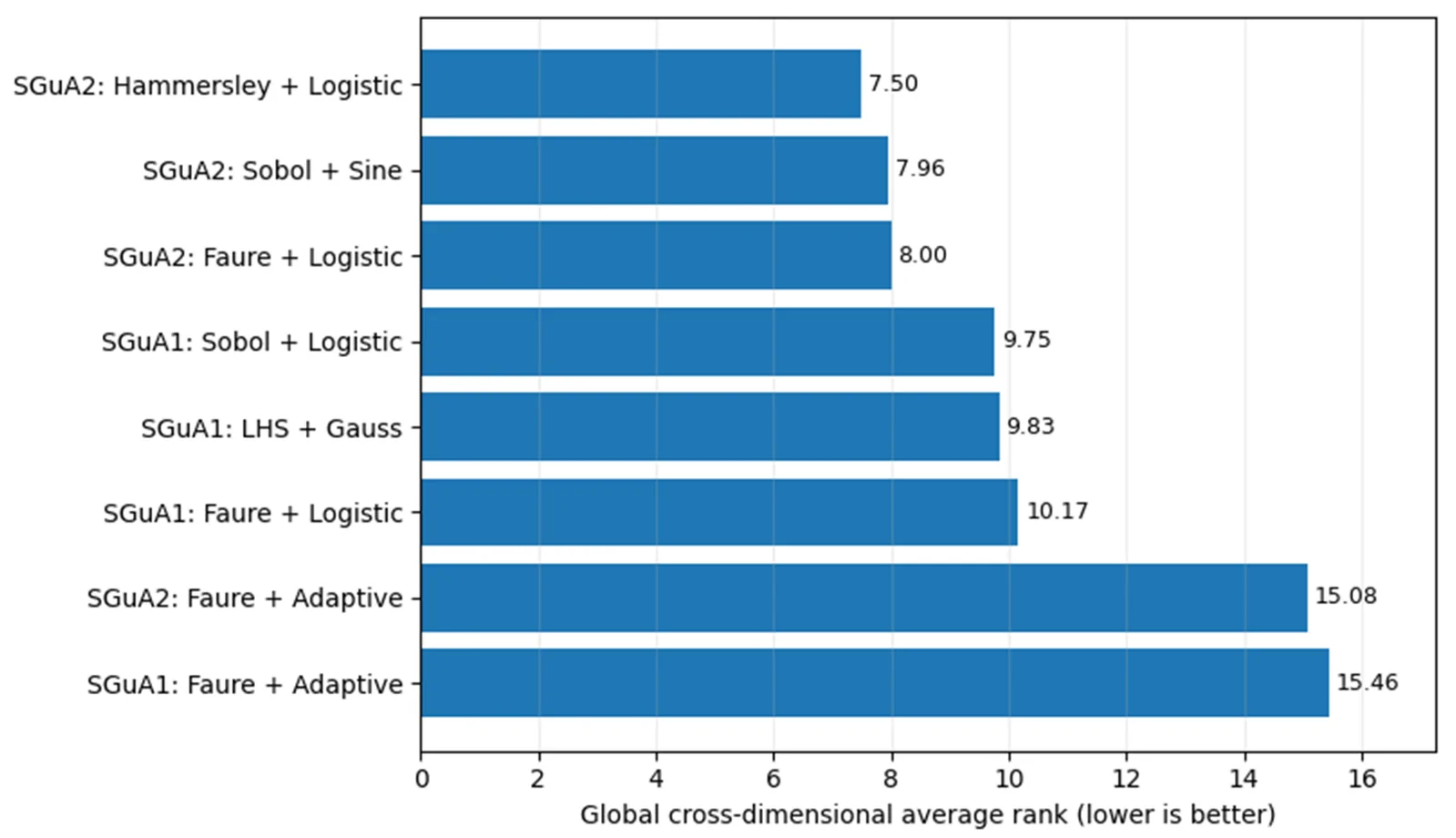
Global cross-dimensional average ranks of the static and adaptive HCQ-SGuA configurations forwarded to Stage 3B. Lower values indicate better performance.

Stage 3A evaluated 30 static HCQ-SGuA configurations obtained by combining the three architecture-specific initialization candidates with five chaotic maps. In addition, one adaptive AOW-HCQ-SGuA configuration was evaluated for each architecture. All candidates were tested on the development functions at D=10 and D=30 using the same screening budget. The global cross-dimensional ranks of the configurations forwarded to the confirmation stage are presented in Figure 4.

For SGuA1, the architecture-specific static ranking identified HCQ-SGuA1[Sobol + Logistic] as the leading configuration, with a static-only average rank of 4.333. HCQ-SGuA1[Faure + Logistic] and HCQ-SGuA1[LHS + Gauss] followed with equal average ranks of 4.542. These three static configurations were retained for high-budget confirmation.

For SGuA2, HCQ-SGuA2[Hammersley + Logistic] achieved the best static-only average rank of 4.042. It was followed by HCQ-SGuA2[Sobol + Sine] and HCQ-SGuA2[Faure + Logistic], with average ranks of 4.417 and 4.542, respectively. These configurations formed the SGuA2 static candidate set for Stage 3B.

As shown in Figure 4, the selected SGuA2 static configurations also obtained the strongest global cross-dimensional ranks, ranging from 7.500 to 8.000. The selected SGuA1 static configurations obtained ranks between 9.750 and 10.167. This result indicates that the strongest SGuA2 combinations generally performed better than their SGuA1 counterparts during the screening stage.

The adaptive variants obtained global average ranks of 15.458 for SGuA1 and 15.083 for SGuA2. Therefore, adaptive map selection did not outperform the leading fixed initialization–map combinations under the Stage 3A budget. Nevertheless, both adaptive candidates were retained for Stage 3B to determine whether their performance improved under a larger function-evaluation budget and new independent seeds.

Logistic appeared in five of the six retained static configurations, confirming its consistent compatibility with both architectures. However, the strongest initialization strategy remained architecture-dependent: Sobol was preferred for SGuA1, whereas Hammersley produced the leading SGuA2 combination. Final configuration selection was therefore deferred to the high-budget confirmation stage.

### 6.5. High-Budget Confirmation and Configuration Selection

Stage 3B re-evaluated the shortlisted static and adaptive configurations using 30 new independent runs and a higher budget of MaxFEs=10,000D. Eight unique candidates were tested across the 12 development functions at D=10 and D=30. Their dimension-specific and cross-dimensional average ranks are summarized in Table 10.

At D=10, HCQ-SGuA2[Sobol + Sine] achieved the best rank. At D=30, HCQ-SGuA2[Faure + Logistic] and HCQ-SGuA1[Sobol + Logistic] shared the leading rank. When both dimensions were considered together, HCQ-SGuA2[Faure + Logistic] obtained the lowest cross-dimensional average rank of 3.292.

Within the SGuA1 architecture, HCQ-SGuA1[Sobol + Logistic] ranked first at both dimensions and was retained as the confirmed factorial champion. Within SGuA2, the dimension-specific ranking changed from Sobol + Sine at D=10 to Faure + Logistic at D=30. Nevertheless, Faure + Logistic produced the strongest aggregate performance and was selected as the confirmed SGuA2 champion.

The confirmation results differed partially from the Stage 3A screening, where Hammersley + Logistic had achieved the strongest SGuA2 rank. This change demonstrates the importance of re-evaluating screened candidates under new seeds and a larger computational budget rather than selecting the final method directly from the initial screening results.

The adaptive variants remained below the strongest static configurations for both architectures. Consequently, adaptive map selection was retained as an ablation configuration but was not selected as the primary method.

Based exclusively on the development-stage confirmation results, HCQ-SGuA2[Faure + Logistic] was designated as the primary proposed configuration. This configuration also corresponds to the direct combination of the independently selected Faure initialization and Logistic chaotic-control champions. The selection was recorded with the status Frozen_Before_Holdout, and no subsequent holdout result was permitted to modify the architecture, initialization strategy, chaotic map, or algorithm parameters.

### 6.6. Performance on Unseen Holdout Functions

The frozen configurations were evaluated on the 17 functions reserved for holdout evaluation at D = 10 and D = 30, using 20 independent runs and a budget of MaxFEs = 5000D. No configuration or parameter was modified after the holdout results were accessed. The architecture-specific ranks and Win–Tie–Loss results of the SGuA2 variants are summarized in Table 11.

W–T–L values are reported from the perspective of HCQ-SGuA2. Average ranks are calculated only across the five SGuA2 configurations included in Table 11.

Table 11 provides a component-wise comparison of the SGuA2 framework under four configurations: the PRNG baseline, Faure-only initialization, Logistic-only control, and Faure + Logistic hybridization. At D = 10, the hybrid configuration achieved the best average rank among the nonadaptive variants (2.294), compared with 2.647 for the PRNG baseline, 2.529 for Faure-only initialization, and 3.294 for Logistic-only control. At D = 30, however, the single-component configurations obtained slightly better ranks, with 2.353 for Faure-only initialization and 2.412 for Logistic-only control, compared with 3.059 for the hybrid configuration. The pairwise differences were not statistically significant after multiple-comparison correction, indicating that the combined effect of Faure initialization and Logistic control is dimension-dependent rather than consistently additive or uniformly superior.

At D=10, HCQ-SGuA2[Faure + Logistic] achieved the best average rank among the SGuA2 variants. It obtained lower mean errors than QR-SGuA2, C-SGuA2, SGuA2-PRNG, and AOW-HCQ-SGuA2 on 10, 11, 10, and 15 of the 17 holdout functions, respectively. These results indicate that the joint use of Faure initialization and Logistic chaotic control was particularly effective at the lower dimensionality.

At D=30, QR-SGuA2 and C-SGuA2 achieved better average ranks than the frozen hybrid configuration. HCQ-SGuA2 recorded six wins against QR-SGuA2, five wins against C-SGuA2, and eight wins against SGuA2-PRNG. However, Holm-adjusted pairwise comparisons showed no statistically significant differences between HCQ-SGuA2 and QR-SGuA2, C-SGuA2, or SGuA2-PRNG at either dimension. Therefore, the observed ranking changes should not be interpreted as conclusive superiority of an individual component configuration.

The clearest result concerned the adaptive variant. HCQ-SGuA2 significantly outperformed AOW-HCQ-SGuA2 at both D=10 and D=30, with Holm-adjusted *p*-values of 0.0085 and 0.0155, respectively. The adaptive method was defeated on 15 of 17 functions at D=10 and 14 of 17 functions at D=30. This finding confirms that the additional map-selection mechanism did not translate into improved generalization on unseen functions.

Overall, the holdout results demonstrate that HCQ-SGuA2 generalized strongly at D=10, while its advantage became less pronounced at D=30. The results also support the pre-specified freezing protocol: although QR-SGuA2 and C-SGuA2 obtained better ranks at D=30, the primary configuration remained HCQ-SGuA2[Faure + Logistic], as required by the development–holdout separation.

### 6.7. Comparison with Established Metaheuristics

**Figure 5:**
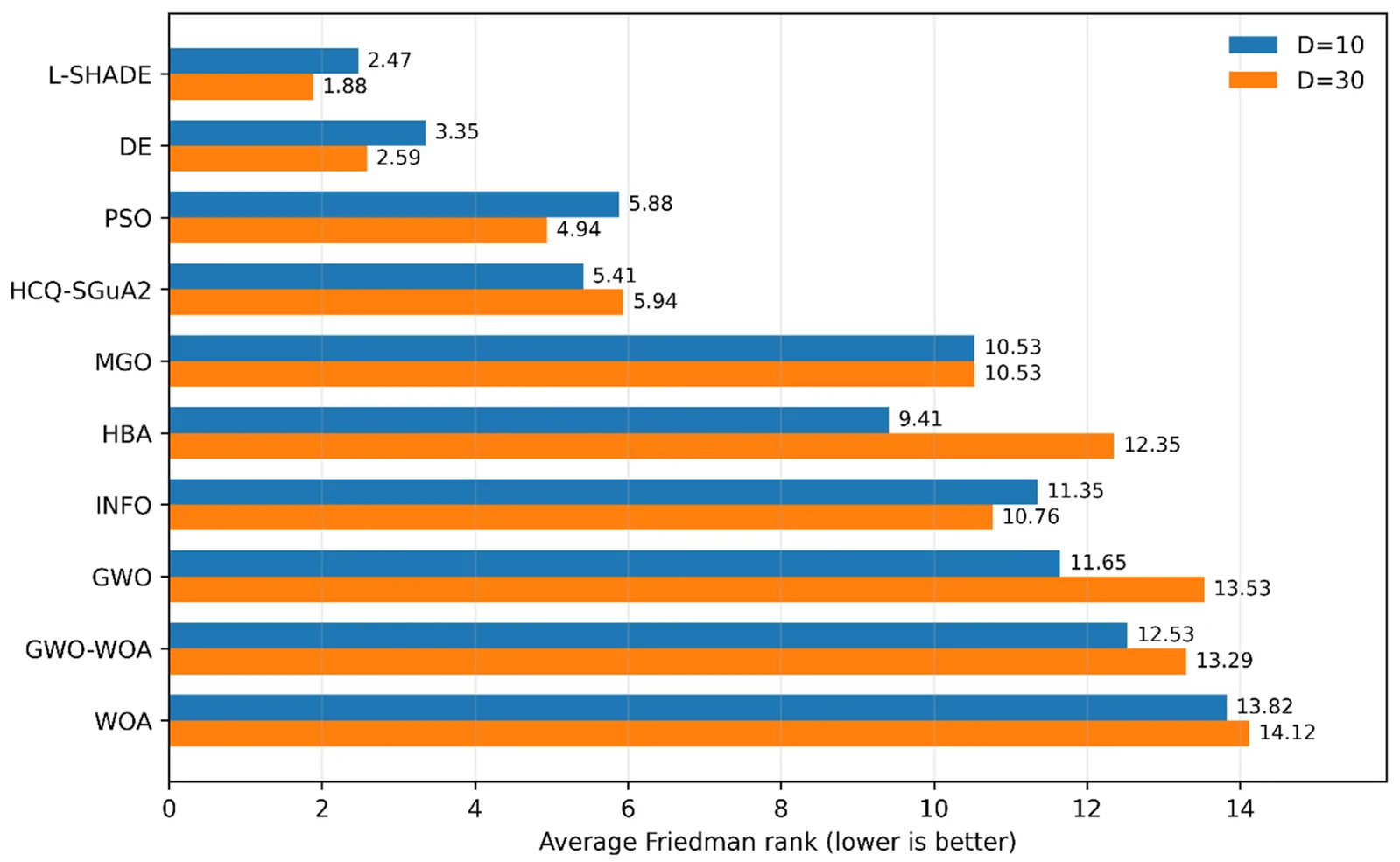
Global average ranks of HCQ-SGuA2 and the external comparator algorithms on the reserved holdout functions. Lower values indicate better performance.

The frozen HCQ-SGuA2[Faure + Logistic] configuration was compared with PSO, DE, GWO, WOA, L-SHADE, HBA, INFO, MGO, and GWO-WOA on the 17 holdout functions. The global average ranks were calculated jointly across all 15 methods included in Stage 4, including the internal SGuA variants. The ranks of the primary method and the external comparators are summarized in Table 12 and illustrated in Figure 5.

The W–T–L values are reported from the perspective of HCQ-SGuA2 against the corresponding comparator. Lower average ranks indicate better performance. The average ranks were calculated jointly across all 15 methods evaluated in Stage 4, including the internal SGuA-derived configurations not individually displayed in this table.

At D=10, HCQ-SGuA2 obtained a global average rank of 5.412 and ranked behind L-SHADE and DE but ahead of PSO and the remaining external comparators. HCQ-SGuA2 outperformed GWO, WOA, INFO, MGO, GWO-WOA, and HBA on at least 13 of the 17 holdout functions. Its comparison with PSO was more balanced, producing nine wins and eight losses.

At D=30, L-SHADE and DE remained the strongest methods, while PSO also achieved a lower average rank than HCQ-SGuA2. Nevertheless, HCQ-SGuA2 outperformed GWO, WOA, HBA, INFO, MGO, and GWO-WOA on all 17 holdout functions. These results indicate that the proposed method maintained strong competitiveness against several swarm- and nature-inspired optimizers as dimensionality increased.

The Friedman tests detected significant differences among the 15 evaluated methods at both D=10 (*p* = 1.32 × 10^−22^) and D=30 (*p* = 4.59 × 10^−35^). Holm-adjusted comparisons showed that L-SHADE and DE significantly outperformed HCQ-SGuA2 at both dimensions. Conversely, HCQ-SGuA2 significantly outperformed GWO, WOA, INFO, MGO, and GWO-WOA at both dimensions. Its difference from HBA was not significant at D=10, but became significant in favor of HCQ-SGuA2 at D=30. No statistically significant difference was found between HCQ-SGuA2 and PSO at either dimension.

Overall, HCQ-SGuA2 did not surpass the advanced differential-evolution methods L-SHADE and DE. However, it achieved competitive performance relative to PSO and exhibited a clear advantage over the other examined nature-inspired comparators. This result positions the proposed framework as a competitive hybrid SGuA variant rather than a universally dominant optimizer.

### 6.8. Convergence and Statistical Analysis

Convergence behavior was evaluated using median best-so-far errors at common function-evaluation checkpoints. CEC2017-F15 at D = 30 was selected before preparing the plot because it is a hybrid landscape, all compared methods produced finite non-degenerate trajectories, and the case displays both early progress and late-stage stagnation. It is presented as an illustrative trajectory only; inferential conclusions are based on all holdout functions and the corrected pairwise tests.

**Figure 6:**
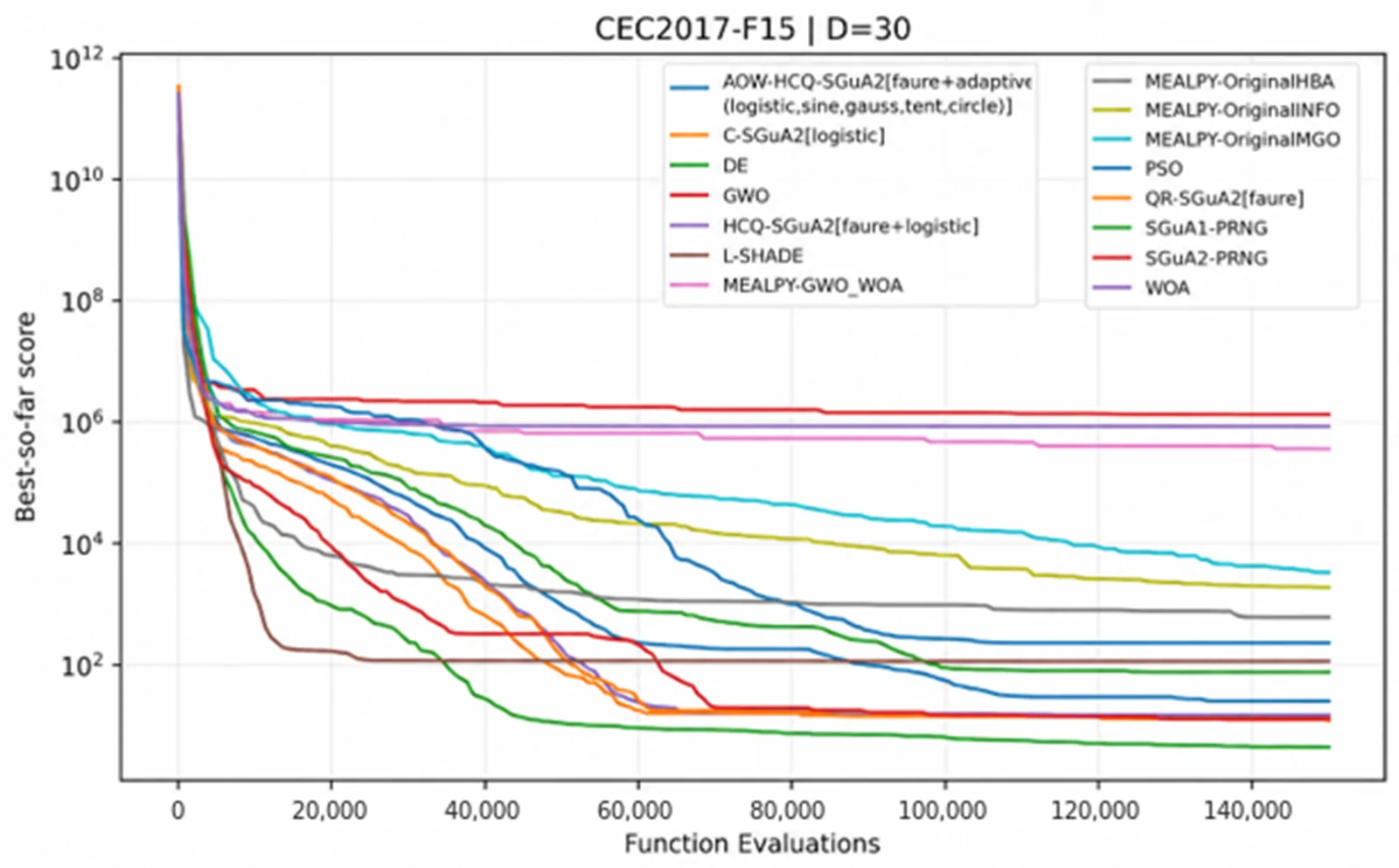
Median best-so-far convergence curves of HCQ-SGuA2 and the compared methods on CEC2017-F15 at D=30.

Figure 6 presents a representative convergence comparison on CEC2017-F15 at D=30. L-SHADE and DE exhibited faster convergence and reached lower final error levels, whereas HCQ-SGuA2 maintained progressive improvement over the evaluation budget and showed more favorable convergence behavior than several weaker comparators, including GWO, WOA, HBA, INFO, MGO, and GWO-WOA. This representative behavior is consistent with the aggregate ranking and pairwise statistical results reported above.

The Friedman test rejected the null hypothesis of equivalent algorithmic performance at both dimensions, with *p* = 1.32 × 10^−22^ for D=10 and *p* = 4.59 × 10^−35^ for D=30. The subsequent Holm-adjusted comparisons confirmed that DE and L-SHADE significantly outperformed HCQ-SGuA2 at both dimensions. Their corresponding $A_{12}$ values were below 0.5, indicating a higher probability of obtaining lower final errors than the proposed method.

In contrast, HCQ-SGuA2 significantly outperformed GWO, WOA, INFO, MGO, and GWO-WOA at both dimensions. At D=30, the effect sizes favoring HCQ-SGuA2 were 0.727 against GWO, 0.751 against WOA, 0.609 against INFO, 0.644 against MGO, and 0.723 against GWO-WOA. HCQ-SGuA2 also significantly outperformed HBA at D=30, with $A_{12}$ = 0.692, although their difference was not significant at D=10.

No significant aggregate difference was found between HCQ-SGuA2 and PSO at either dimension. Similarly, its differences from QR-SGuA2, C-SGuA2, and the PRNG-based SGuA configurations were not statistically significant after Holm correction. These results indicate that the Faure–Logistic hybridization provided a competitive but problem-dependent contribution rather than uniform superiority over all internal configurations.

The statistical and convergence findings therefore lead to a consistent conclusion: HCQ-SGuA2 substantially improves upon several established nature-inspired optimizers and the adaptive HCQ variant, while advanced differential-evolution methods remain stronger overall. This balanced outcome supports the proposed framework as a competitive SGuA-based optimizer without implying universal dominance.

### 6.9. Constrained Engineering Design Results

The practical performance of the frozen HCQ-SGuA2[Faure + Logistic] configuration was evaluated on the tension/compression spring, pressure vessel, and welded beam design problems. Each method was executed for 30 independent runs using N=100 and MaxFEs=100,000. All algorithms produced feasible solutions in every run, indicating that the applied constraint-handling procedure reliably preserved feasibility. The principal results are summarized in Table 13.

For the tension/compression spring problem, HCQ-SGuA2 obtained a best objective value of 0.012699, which was close to the best values achieved by DE and L-SHADE. However, its higher mean and standard deviation indicate lower run-to-run consistency. The adaptive and quasi-random SGuA2 variants also achieved better mean results than the frozen hybrid configuration on this problem.

On the pressure vessel problem, HCQ-SGuA2 produced the strongest mean performance among the SGuA-derived configurations. It outperformed AOW-HCQ-SGuA2, QR-SGuA2, C-SGuA2, and both PRNG-based architectures. Nevertheless, DE, L-SHADE, GWO, and PSO achieved lower mean objective values. The discrete thickness restrictions were satisfied, and all HCQ-SGuA2 runs remained feasible.

For the welded beam problem, the best HCQ-SGuA2 solution of 1.725335 was close to the best value of 1.724852 obtained by DE and L-SHADE. Its mean result was better than those of QR-SGuA2, C-SGuA2, SGuA1-PRNG, SGuA2-PRNG, and WOA, although the adaptive HCQ configuration achieved a lower mean value. The relatively large standard deviation again shows that HCQ-SGuA2 occasionally approached the best-known region but did not do so consistently across all runs.

Overall, the engineering experiments demonstrate that HCQ-SGuA2 can generate feasible and near-best solutions for all three constrained problems. Its main limitation is not solution feasibility but consistency, particularly when compared with DE and L-SHADE. The findings suggest that further improvements should focus on constraint-aware local refinement and stronger late-stage exploitation.

### 6.10. Quantitative Performance-Magnitude and Runtime Assessment

To complement the rank-based and significance-test summaries, Table 14 quantifies how large the observed objective-value differences were and reports the corresponding mean execution times. The comparison covers the reserved CEC2017 holdout functions at D = 10 and D = 30 and the three constrained engineering-design problems; it therefore distinguishes statistical ordering from practical performance magnitude and computational cost.

The magnitude analysis supports the deliberately moderated claims. At D = 10, HCQ-SGuA2 achieved positive mean relative advantages over the component-only SGuA2 variants and over most swarm- and nature-inspired comparators, but it remained behind DE, L-SHADE, and PSO. At D = 30, the negative gaps against QR-SGuA2, C-SGuA2, the PRNG SGuA variants, DE, L-SHADE, and PSO confirm that the hybrid advantage was not dimensionally uniform; in contrast, large positive gaps remained against GWO, WOA, HBA, INFO, MGO, GWO-WOA, and the adaptive HCQ variant. The engineering results show smaller and problem-dependent differences. Runtime comparisons likewise reveal no universal speed advantage: HCQ-SGuA2 was faster than several advanced or hybrid comparators but slower than some simpler methods. These findings complement, rather than replace, the Holm-corrected statistical comparisons and support describing HCQ-SGuA2 as competitive rather than universally superior.

### 6.11. Overall Discussion

The multi-stage results show that the performance of HCQ-SGuA depends on the interaction among architecture, initialization strategy, chaotic controller, dimensionality, and problem structure. Stage 0 did not reveal a universally superior SGuA architecture. SGuA1 achieved the better aggregate diagnostic rank, whereas SGuA2 exhibited advantages on selected higher-dimensional functions. Accordingly, both architectures were retained during component screening to account for their dimension-dependent performance characteristics.

The initialization experiments similarly demonstrated that structured sampling does not guarantee uniform improvement over PRNG initialization. Faure achieved the strongest cross-dimensional performance for both architectures, while Sobol, Latin hypercube, and Hammersley remained competitive under specific architecture–dimension combinations. This result suggests that the value of low-discrepancy initialization is determined not only by population uniformity but also by how the resulting population interacts with the subsequent search operators. This problem- and algorithm-dependent behavior is consistent with previous studies showing that the effectiveness of population initialization varies across optimization methods, problem landscapes, and dimensional settings [17,22,23].

Among the chaotic controllers, Logistic provided the most consistent aggregate performance across both architectures. In contrast, Tent and Circle generally produced weaker results. The differences among the maps indicate that replacing pseudo-random control with a chaotic sequence is not inherently beneficial. The statistical and distributional properties of the selected map must be compatible with the decision mechanisms of the optimization algorithm. A similar map-dependent behavior has been reported in chaotic metaheuristic studies, where the effectiveness of chaotic integration varied according to the selected map and the underlying search mechanism [24,25,26].

The static hybrid-screening results further confirmed that independently strong components do not always form the best combination. Although Faure and Logistic were identified as the best-performing individual components, other combinations, including Hammersley–Logistic and Sobol–Sine, achieved stronger performance during the initial hybrid-screening stage. However, their relative positions changed under the larger confirmation budget and new seeds. This variation highlights the importance of an independent confirmation stage before final configuration selection.

HCQ-SGuA2[Faure + Logistic] achieved the best aggregate rank during high-budget confirmation and was therefore frozen before the holdout evaluation. Its performance on the reserved holdout functions was strongest at D=10, whereas QR-SGuA2 and C-SGuA2 obtained better average ranks at D=30. These differences were not statistically significant after multiple-comparison correction. Consequently, the holdout findings support the competitiveness of the frozen hybrid configuration but do not establish uniform superiority over its individual initialization and chaotic-control components.

Against the external comparators, HCQ-SGuA2 significantly outperformed several swarm-based and nature-inspired methods, including GWO, WOA, INFO, MGO, and GWO-WOA. Its performance was statistically comparable with PSO but remained below DE and L-SHADE. The stronger results of DE and L-SHADE indicate the continued effectiveness of differential-evolution-based search on these functions, with L-SHADE additionally benefiting from success-history parameter adaptation and linear population-size reduction [76,79].

The engineering-design experiments demonstrated that HCQ-SGuA2 consistently produced feasible solutions and approached the best-performing methods on all three problems. However, its larger standard deviations showed that near-optimal solutions were not reached with the same consistency as DE and L-SHADE. Therefore, the principal practical limitation of the proposed method is not constraint satisfaction but late-stage exploitation and run-to-run stability.

An additional limitation arises from the benchmark implementation used in this study. The experiments were conducted using the CEC2017 function classes F1–F29 available in OPFUNU 1.0.4. This implementation-defined set includes F2 and does not include F30, whereas the official CEC2017 competition subset excluded F2 because of its reported numerical instability and retained F30 [73]. Consequently, the reported rankings and statistical comparisons should be interpreted as being specific to the employed OPFUNU-based function set. Direct numerical comparison with studies using the official F1 and F3–F30 subset should therefore be made cautiously.

Overall, the results support HCQ-SGuA2 as a competitive SGuA-based optimization framework rather than a universally dominant metaheuristic. Its main contribution lies in the architecture-aware and data-driven selection of initialization and chaotic-control components, together with a development–confirmation–holdout protocol that reduces configuration-selection bias. Future improvements should focus on adaptive mechanisms with lower control overhead, constraint-aware local refinement, and more effective exploitation in high-dimensional and engineering-design problems.

## 7. Conclusions

This study introduced HCQ-SGuA as an architecture-aware framework that combines structured Sowing with chaotic control of the biologically framed Mating, Branching, and Vaccinating mechanisms. The proposed framework systematically evaluates the effects of architecture, initialization, chaotic control, and their interaction through a multi-stage development, confirmation, and holdout protocol.

Faure initialization and Logistic chaotic control emerged as the most consistent individual components, while high-budget confirmation identified HCQ-SGuA2[Faure + Logistic] as the final configuration. On the holdout functions, this configuration remained competitive with its individual components, although the observed differences were not statistically significant and varied with dimensionality. HCQ-SGuA2 outperformed several swarm- and nature-inspired comparators, achieved performance comparable to PSO, and remained below DE and L-SHADE. The engineering-design experiments further demonstrated that the method can consistently obtain feasible and competitive best-run solutions, although its run-to-run stability was lower than that of the leading differential-evolution methods.

The adaptive operator-weighted variant did not outperform the strongest static configurations during screening, confirmation, or holdout evaluation. Under the investigated settings, the additional flexibility of adaptive map selection therefore provided no consistent performance advantage over fixed chaotic control, indicating that the effectiveness of adaptive hybridization is strongly dependent on the underlying search architecture and computational setting.

The findings of this study are limited to D = 10 and D = 30, the OPFUNU 1.0.4 implementation of CEC2017 F1–F29, three classical engineering-design problems, and the adaptive-control settings considered in the experiments. Future research should extend the evaluation to higher-dimensional settings, the official CEC2017 F1/F3–F30 suite, distribution-matched chaotic controls, broader parameter-sensitivity analyses, and additional constrained engineering-design problems. Further methodological development may also focus on improving late-stage exploitation, run-to-run stability, and the computational efficiency of adaptive control mechanisms.

## Figures and Tables

**Table 1:** Symbols, abbreviations, and notation used throughout the manuscript.

Symbol/Abbreviation	Definition
SGuA/SGuA1/SGuA2	Saplings Growing Up Algorithm and its collective-selection/sequential-selection architectures
HCQ-SGuA	Hybrid Chaotic–Quasi-Random SGuA
QR-SGuA/C-SGuA	Initialization-only and chaotic-control-only SGuA configurations
AOW-HCQ-SGuA	Adaptive Operator-Weighted HCQ-SGuA
PRNG/LHS	Pseudo-random number generator/Latin Hypercube Sampling
*N*/*D*	Population size/problem dimension
G(t), xi	Garden (population) at iteration t/its *i*-th candidate solution
f(x), gr(x)	Objective function/*r*-th inequality constraint
Fitness/S(x)	Scalar candidate score used for comparison and elitist selection
FEs/MaxFEs	Consumed/maximum permitted objective-function evaluations
W–T–L	Win–Tie–Loss counts from the perspective of the primary HCQ-SGuA configuration
Δrel	Mean normalized relative objective-value difference; positive values favor HCQ-SGuA2 and negative values favor the comparator
tHCQ/tcomp	Mean execution time per run for HCQ-SGuA2/the corresponding comparator (seconds)
zk	Normalized auxiliary state of the chaotic controller at controller step *k*; it is not an optimization decision variable
fC (⋅)	Nonlinear state-update function associated with chaotic map *C*
k	Internal update-step index of the chaotic controller; distinct from the optimization iteration index *t*
$f_{bias}$	Function-specific additive bias of the corresponding CEC2017 benchmark function.
$E\left(x\right)$	Bias-corrected objective error used for reporting the CEC2017 results
$D_{c}$	Mean coil diameter in the tension/compression spring design problem

**Table 2:** Methodological distinction among the SGuA-derived configurations.

Configuration	Architecture	Initialization	Operator Control
Reference SGuA	A	PRNG	PRNG
QR-SGuA	A	Q	PRNG
C-SGuA	A	PRNG	Fixed C
Static HCQ-SGuA	A	Q	Fixed C
AOW-HCQ-SGuA	A	Q	Adaptive chaotic-map pool

**Table 3:** Development and holdout partition of the OPFUNU-implemented CEC2017 F1–F29 function set.

Partition	Function IDs	Number	Experimental Purpose
Development	F1, F3, F4, F7, F9, F10, F13, F16, F19, F20, F24, F28	12	Architecture and component screening, hybrid construction, and confirmation
Holdout	F2, F5, F6, F8, F11, F12, F14, F15, F17, F18, F21, F22, F23, F25, F26, F27, F29	17	Frozen final evaluation

**Table 4:** Multi-stage experimental protocol used for the development and evaluation of HCQ-SGuA.

Stage	Main Purpose	Evaluated Configurations	Stage Output
Stage 0	Architecture diagnosis	SGuA1-PRNG and SGuA2-PRNG	Architecture-level diagnostic results
Stage 1	Initialization screening	Eight initialization strategies on both architectures	Ranked initialization strategies for each architecture
Stage 2	Chaotic-control screening	PRNG control and five chaotic maps on both architectures	Ranked chaotic-control strategies for each architecture
Stage 3A	Hybrid screening	Static factorial and adaptive hybrid configurations	Shortlisted hybrid candidates
Stage 3B	High-budget confirmation	Shortlisted static, sequential, and adaptive candidates	Frozen primary configuration
Stage 4	Unseen holdout evaluation	Frozen SGuA configurations and comparison algorithms	Final numerical benchmark results
Stage 5	Engineering validation	Frozen configurations and comparison algorithms	Constrained engineering-design results

**Table 5:** Comparator algorithms used in the experimental evaluation.

Algorithm	Abbreviation	Main Search Mechanism	Method Category
Particle Swarm Optimization	PSO	Personal- and global-best-guided velocity updating	Swarm intelligence
Differential Evolution	DE	Differential mutation, crossover, and selection	Evolutionary computation
Grey Wolf Optimizer	GWO	Hierarchical leadership and prey encircling	Swarm intelligence
Whale Optimization Algorithm	WOA	Encircling, spiral movement, and random exploration	Swarm intelligence
Linear Population Size Reduction SHADE	L-SHADE	Success-history adaptation and linear population reduction	Adaptive evolutionary computation
Honey Badger Algorithm	HBA	Digging- and honey-finding-based exploration and exploitation	Swarm intelligence
Weighted Mean of Vectors Optimizer	INFO	Weighted-mean updating, vector combination, and local search	Mathematical optimization
Mountain Gazelle Optimizer	MGO	Social hierarchy, herd behavior, and movement-based search	Swarm intelligence
Grey Wolf–Whale Optimization Algorithm	GWO-WOA	Hybridization of grey-wolf leadership and whale-inspired updating	Hybrid swarm intelligence

**Table 6:** Common and method-specific parameter settings used in the experiments.

Algorithm/Component	Parameter	Value or Setting
All algorithms	Initial population size	N=100
All algorithms	Stopping criterion	Strict MaxFEs
All algorithms	Search bounds	Function-specific
All algorithms	Run seeds	Identical within each experimental condition
All algorithms	Initial evaluations	Included in MaxFEs
SGuA variants	Mating component probability	$p_{c}=0.50$
SGuA variants	Branching probability	$p_{b}=0.40$
SGuA variants	Branching power	β=0.10
SGuA variants	Maximum vaccinating coefficient	$\gamma_{max}=0.60$
Chaotic controllers	Initial state	$x_{0}\sim U(0.05,0.95)$
Chaotic controllers	Burn-in length	1000 iterations
AOW-HCQ-SGuA	Exploration probability	ε=0.10
AOW-HCQ-SGuA	Softmax temperature	τ=0.35
AOW-HCQ-SGuA	Score learning rate	α=0.20
AOW-HCQ-SGuA	Stagnation patience	12 generations
AOW-HCQ-SGuA	Refresh fraction	ρ=0.10
PSO	Inertia weight	Linearly decreased from 0.90 to 0.40
PSO	Acceleration coefficients	$c_{1}=c_{2}=2.0$
DE	Mutation strategy	DE/rand/1/bin
DE	Scale factor	F=0.50
DE	Crossover probability	CR=0.90
GWO	Control parameter	Linearly decreased from 2 to 0
WOA	Control parameter	Linearly decreased from 2 to 0
L-SHADE	Historical memory size	H=6
L-SHADE	Minimum population size	4
L-SHADE	Population schedule	Linear reduction from N=100
HBA, INFO, MGO, GWO-WOA	Implementation and algorithm-specific parameters	MEALPY v3.0.3; native default parameters retained; N=100; termination controlled by strict MaxFEs

**Table 7:** Architecture diagnostic results on the development functions.

Dimension	SGuA1 Average Rank	SGuA2 Average Rank	Mean-Error Wins, SGuA1/SGuA2	Significant Wins, SGuA1/SGuA2
D=10	1.333	1.667	8/4	0/0
D=30	1.500	1.500	6/6	0/2
Overall	1.417	1.583	14/10	0/2

**Table 8:** Architecture-specific average ranks of the leading initialization strategies.

Architecture	Initialization Strategy	D=10	D=30	Overall Average Rank	Selection Status
SGuA1	Faure	**2.833**	4.417	**3.625**	Retained—champion
SGuA1	Sobol	4.083	**3.500**	3.792	Retained
SGuA1	Latin hypercube	4.000	4.167	4.083	Retained
SGuA1	PRNG	5.250	3.583	4.417	Reference
SGuA2	Faure	3.583	**3.667**	**3.625**	Retained—champion
SGuA2	Sobol	**3.250**	4.250	3.750	Retained
SGuA2	Hammersley	4.333	4.500	4.417	Retained
SGuA2	PRNG	4.417	3.833	4.125	Reference

Note: Bold values indicate the lowest (best) average rank within each architecture for the corresponding dimension or the overall average-rank column.

**Table 9:** Architecture-specific average ranks obtained in the chaotic-control screening.

Architecture	Control Source	D=10	D=30	Overall Average Rank	Status
SGuA1	Logistic	**2.000**	**2.417**	**2.208**	Champion
SGuA1	Gauss	2.250	2.750	2.500	Candidate
SGuA1	Sine	2.583	**2.417**	2.500	Candidate
SGuA1	PRNG control	3.167	**2.417**	2.792	Reference
SGuA1	Tent	5.083	5.167	5.125	Candidate
SGuA1	Circle	5.917	5.833	5.875	Candidate
SGuA2	Logistic	2.333	**2.000**	**2.167**	Champion
SGuA2	PRNG control	3.083	**2.000**	2.542	Reference
SGuA2	Gauss	2.583	2.750	2.667	Candidate
SGuA2	Sine	**2.083**	3.250	2.667	Candidate
SGuA2	Tent	5.000	5.083	5.042	Candidate
SGuA2	Circle	5.917	5.917	5.917	Candidate

Note: Bold values indicate the lowest (best) average rank within each architecture for the corresponding dimension or the overall average-rank column. When multiple control sources share the lowest rank, all tied values are shown in bold.

**Table 10:** Global average ranks obtained in the high-budget confirmation stage.

Architecture	Configuration	D=10	D=30	Cross-Dimensional Rank
SGuA2	Faure + Logistic	3.583	**3.000**	**3.292**
SGuA1	Sobol + Logistic	3.833	**3.000**	3.417
SGuA2	Sobol + Sine	**3.083**	4.333	3.708
SGuA2	Hammersley + Logistic	3.917	3.667	3.792
SGuA1	Faure + Logistic	4.750	4.583	4.667
SGuA1	LHS + Gauss	4.667	5.417	5.042
SGuA2	Faure + Adaptive	5.500	5.000	5.250
SGuA1	Faure + Adaptive	6.667	7.000	6.833

Note: Bold values indicate the lowest (best) average rank among all evaluated configurations for the corresponding dimension or the cross-dimensional rank. When multiple configurations share the lowest rank, all tied values are shown in bold.

**Table 11:** Performance of the SGuA2 variants on the reserved holdout functions.

Configuration	Rank, D=10	W–T–L, D=10	Rank, D=30	W–T–L, D=30
HCQ-SGuA2[Faure + Logistic]	**2.294**	—	3.059	—
C-SGuA2[Logistic]	2.529	11–0–6	2.412	5–0–12
QR-SGuA2[Faure]	2.647	10–0–7	**2.353**	6–0–11
SGuA2-PRNG	3.294	10–0–7	2.588	8–0–9
AOW-HCQ-SGuA2	4.235	15–0–2	4.588	14–0–3

Note: Bold values indicate the lowest (best) average rank among the five SGuA2 configurations for the corresponding dimension.

**Table 12:** Holdout performance of HCQ-SGuA2 and the external comparator algorithms.

Algorithm	Rank, D=10	Rank, D=30	Overall Rank	W–T–L, D=10	W–T–L, D=30
L-SHADE	**2.471**	**1.882**	**2.176**	1–0–16	2–0–15
DE	3.353	2.588	2.971	3–0–14	2–0–15
PSO	5.882	4.941	5.412	9–0–8	6–0–11
HCQ-SGuA2	5.412	5.941	5.676	—	—
MGO	10.529	10.529	10.529	15–0–2	17–0–0
HBA	9.412	12.353	10.882	13–0–4	17–0–0
INFO	11.353	10.765	11.059	16–0–1	17–0–0
GWO	11.647	13.529	12.588	16–0–1	17–0–0
GWO-WOA	12.529	13.294	12.912	16–0–1	17–0–0
WOA	13.824	14.118	13.971	16–0–1	17–0–0

Note: Bold values indicate the lowest (best) average rank among the algorithms displayed in the table for the corresponding dimension or the overall-rank column.

**Table 13:** Performance of HCQ-SGuA2 on the constrained engineering design problems.

Engineering Problem	HCQ-SGuA2 Mean ± Std	Best HCQ-SGuA2 Solution	Rank	Best-Performing Method	Best Method Mean ± Std
Tension/compression spring	0.013915 ± 0.001224	0.012699	8/11	L-SHADE	0.012665 ± 2.08 × 10^−17^
Pressure vessel	6296.3978 ± 241.9866	6062.4145	5/11	DE	5850.3831 ± 1.85 × 10^−12^
Welded beam	1.931767 ± 0.250318	1.725335	6/11	DE/L-SHADE	1.724852 ± 2.26 × 10^−16^

**Table 14:** Quantitative performance magnitude and computational-time comparison of HCQ-SGuA2 with competing algorithms.

Algorithm	CEC2017, D = 10 Δ_rel_ (%)	W/T/L	Runtime HCQ/Comp. (s)	CEC2017, D = 30 Δ_rel_ (%)	W/T/L	Runtime HCQ/Comp. (s)	Engineering Problems Δ_rel_ (%)	W/T/L	**Runtime HCQ/Comp. (s)**
AOW-HCQ-SGuA2	**15.24**	15/0/2	4.236/4.366	**20.17**	14/0/3	19.906/20.717	−1.40	1/0/2	3.723/3.879
C-SGuA2[Logistic]	**2.39**	11/0/6	4.236/4.233	−9.71	5/0/12	19.906/19.890	**2.27**	3/0/0	3.723/3.718
QR-SGuA2[Faure]	**0.67**	10/0/7	4.236/4.057	−12.28	6/0/11	19.906/18.430	**2.58**	2/0/1	3.723/3.510
SGuA1-PRNG	**15.94**	12/0/5	4.236/4.087	−3.59	9/0/8	19.906/18.536	**3.29**	3/0/0	3.723/3.517
SGuA2-PRNG	**8.29**	10/0/7	4.236/4.054	−7.81	8/0/9	19.906/18.423	**4.06**	3/0/0	3.723/3.506
DE	−51.42	3/0/14	4.236/4.526	−62.14	2/0/15	19.906/19.963	−8.93	0/0/3	3.723/4.338
GWO	**69.51**	16/0/1	4.236/4.558	**81.29**	17/0/0	19.906/20.110	−8.44	0/0/3	3.723/4.672
L-SHADE	−67.14	1/0/16	4.236/6.109	−67.06	2/0/15	19.906/25.697	−8.70	0/0/3	3.723/7.841
GWO-WOA	**68.70**	16/0/1	4.236/5.307	**81.49**	17/0/0	19.906/23.407	—	—	—
HBA	**31.33**	13/0/4	4.236/5.526	**75.35**	17/0/0	19.906/24.190	—	—	—
INFO	**57.06**	16/0/1	4.236/7.726	**60.52**	17/0/0	19.906/31.364	—	—	—
MGO	**55.96**	15/0/2	4.236/4.928	**65.62**	17/0/0	19.906/21.733	—	—	—
PSO	−19.17	9/0/8	4.236/3.435	−16.02	6/0/11	19.906/16.452	−6.09	0/0/3	3.723/2.241
WOA	**76.60**	16/0/1	4.236/3.816	**90.79**	17/0/0	19.906/17.708	**5.07**	2/0/1	3.723/3.075

Table note: HCQ denotes HCQ-SGuA2[Faure + Logistic], the primary proposed method. Δ_rel_ represents the mean normalized relative objective-value difference between the comparator and HCQ across the corresponding problems and is calculated as $100(fcomp-fHCQ)/max(\left|fcomp\right|,\;\left|fHCQ\right|,10^{-12})$. Since all problems are formulated as minimization problems, positive Δ_rel_ values indicate an advantage for HCQ, whereas negative values indicate an advantage for the comparator. W/T/L denotes the number of problems in which HCQ obtained a lower/equal/higher mean objective value than the comparator. Runtime values are reported as HCQ/comparator mean execution time per run in seconds. CEC2017 comparisons comprise 17 holdout functions with 20 independent runs per function; the engineering comparison comprises three design problems with 30 independent runs per problem. Bold values indicate the most favorable result in each row.

## Data Availability

The benchmark partition, run seeds, parameter settings, mathematical formulations, source code, configuration files, diagnostic outputs, and raw run-level results supporting this study are publicly available in the HCQ-SGuA reproducibility repository at https://github.com/ebuseyy/HCQ-SGuA-Document (accessed on 9 September 2026). Additional methodological details and engineering problem formulations are provided in the article and Supplementary Materials.

## Supplementary Materials

The following supporting information can be downloaded at https://www.mdpi.com/article/10.3390/biomimetics11090663/s1, Algorithm S1–S6; Table S1 and Table S2; Sections S1–S5.
