# Supplementary material for: HCQ-SGuA: A Plant-Growth-Inspired Hybrid Saplings Growing Up Algorithm with Quasi-Random Initialization and Chaotic Operator Control for Continuous Optimization
Source: Biomimetics (Basel). 2026 Sep 16;11(9):663. doi: 10.3390/biomimetics11090663 (PMC13604883; doi:10.3390/biomimetics11090663)
Supplement: Supplementary file 1 [file biomimetics-11-00663-s001.zip › biomimetics-4521689-supplementary.pdf]

biomimetics

MDPI

Supplementary Material for HCQ-SGuA

Ebubekir SEYYARER

This file contains material moved from the main manuscript to improve focus and readability while preserving complete methodological reproducibility.

Section S1. Original SGuA Pseudocodes

Notation: Fitness denotes the scalar candidate score $S(x)$ defined in Section 4.2.1 of the main manuscript; MaxFEs is the maximum permitted number of objective/constraint evaluations, and FEs is the number already consumed.

Supplementary Algorithm S1 Original Sowing procedure of SGuA

Input: Problem dimension (n), lower bounds, upper bounds, maximum population size
Output: Initial garden (population) P
1: // G is a garden, I is an index set, and I_e is an extended index set.
2: Create two initial saplings: G[1] containing the lower bounds and G[2] containing the upper bounds of the branches.
3: index ← 3
4: k ← 2
5: While P is not full do
6: Let i_e be an element of I_e, where each i_e is extended with a bit value corresponding to the partition.
7: i ← 1
8: While P is not full and not all saplings are generated for a specific k ($i \le 2^k-2$) do
9: i is a k-bit number and i_e corresponds to the extended value of i.
10: Each bit of i is extended up to the length of the corresponding part of G[1] and G[2].
11: For j ← 1 to n do
12: r ← random(0, 1) // uniformly distributed real number
13: If the j-th bit of i_e == 1 then
14: j-th branch of P[index] ← G[1] * r
15: Else
16: j-th branch of P[index] ← G[2] * r
17: End If
18: End For
19: index ← index + 1
20: i ← i + 1
21: End While
22: k ← k + 1
23: End While

Notation note: i_e denotes the active coordinate index and I_e denotes the corresponding index set used by the interval-partitioning loop.

Supplementary Algorithm S2. Mating Operator

Input: Two saplings $S_1 = \{s_{1,1}, s_{1,2}, \ldots, s_{1,n}\}$ and $S_2 = \{s_{2,1}, s_{2,2}, \ldots, s_{2,n}\}$, problem dimension (n), normalization factor (R)

Output: Updated saplings S_1 and S_2

1. j←1, …, n
2. Compute $P_m(S_1,S_2) = 1-\frac{(\sum_{j=1}^{n}(s_{1,j}-s_{2,j})^2)^{1/2}}{R}$
3. i←1, …, n
4. if $P_m(S_1,S_2)$≥random(0,1) then

$S_1 \leftarrow S_1 - s_{1,i}$ and $S_2 \leftarrow S_2 - s_{2,i}$, $S_1 \leftarrow S_1 + s_{2,i}$ and $S_2 \leftarrow S_2 + s_{1,i}$

Supplementary Algorithm S3. Branching Operator

Input: A sapling $S_1 = \{s_{1,1}, s_{1,2}, \ldots, s_{1,n}\}$, problem dimension (n)

Output: Updated (branched) sapling S_1

1. i←1, …, n
2. j←i+1, …, n
3. if no branch then $P(s_{1,j}|s_{1,i})=1$

and branching operation is applied.

4. else

$P(s_{1,j}|s_{1,i})=1-\frac{1}{(|j-i|)^2}$, i≠j

Or

$P(s_{1,j}|s_{1,i})=1-\frac{1}{e^{(|j-i|)^2}}$

5. if $P(s_{1,j}|s_{1,i})$≥random(0,1) then $s_{1,j}$ will be a branch.

Supplementary Algorithm S4. Vaccinating Operator

Input: Two saplings $S_1 = \{s_{1,1}, s_{1,2}, \ldots, s_{1,n}\}$ and $S_2 = \{s_{2,1}, s_{2,2}, \ldots, s_{2,n}\}$ problem dimension (n)

Output: Vaccinated saplings S'_1 and S'_2

1. i←1, …, n
2. $Dis(S_1, S_2)=\sum_{i=1}^{n} s_{1,i} \oplus s_{2i}$
3. if $Dis(S_1, S_2)$≥random then
4. $S'_1 \begin{cases} s_{1i} & \text{if } s_{1i} = s_{2i} \\ \text{random}(1) & \text{if } s_{1i} \neq s_{2i} \end{cases}$

And

$S'_2 \begin{cases} s_{2i} & \text{if } s_{2i} = s_{1i} \\ \text{random}(1) & \text{if } s_{2i} \neq s_{1i} \end{cases}$

Supplementary Algorithm S5. Saplings Growing up Algorithm (SGuA1 Architecture)

Input: Objective function, termination criterion based on Maximum Function Evaluations (MaxFEs), population size

Output: The best sapling (optimal solution) found in the garden

1. t←0 // Initialize the generation counter
2. Sowing_Phase(G(t)) // Initialize the garden (initial population)
3. Calculate_Fitness(G(t))
4. While the termination criteria are not met do

4.1. $G_1(t) \leftarrow$ Mating(G(t))

4.2. $G_2(t) \leftarrow$ Branching(G(t))

4.3. $G_3(t) \leftarrow$ Vaccinating(G(t))

4.4. Evaluate the fitness values of the newly generated temporary saplings $(G_1(t) \cup G_2(t) \cup G_3(t))$

4.5. $G(t+1) \leftarrow$ Select $(G(t) \cup G_1(t) \cup G_2(t) \cup G_3(t))$

4.6. t←t+1

Supplementary Algorithm S6. Saplings Growing up Algorithm (SGuA2 Architecture)

Input: Objective function, termination criterion based on Maximum Function Evaluations (MaxFEs), population size

Output: The best sapling (optimal solution) found in the garden

1. t←0 // Initialize the generation counter

2. Sowing_Phase (G(t)) // Initialize the garden (initial population)

3. Calculate_Fitness (G(t))

4. While the termination criteria are not met do

4.1. $G_1(t) \leftarrow$ Mating (G(t))

4.2. Calculate_Fitness ($G_1(t)$)

4.3. $G_m(t) \leftarrow$ Select ($G_1(t) \cup G(t)$)

4.4. $G_2(t) \leftarrow$ Branching ($G_m(t)$)

4.5. Calculate_Fitness ($G_2(t)$)

4.6. $G_b(t) \leftarrow$ Select ($G_2(t) \cup G_m(t)$)

4.7. $G_3(t) \leftarrow$Vaccinating ($G_b(t)$)

4.8. Calculate_Fitness ($G_3(t)$)

4.9. $G(t+1) \leftarrow$Select ($G_3(t) \cup G_b(t)$)

4.10. t←t+1

Section S2. Population-Initialization Definitions and Applications

Supplementary Table S1. Main characteristics of the population-initialization strategies considered in the study.

Strategy	Class	Dimension	Problem	Ref.
PRNG	Stochastic sampling	Multidimensional	Independent uniform sampling	[23,34]
Latin Hypercube Sampling	Stratified space-filling sampling	Multidimensional	One sample from each marginal stratum	[22,33]
Van Der Corput	Low-discrepancy sequence	One-dimensional	Radical-inverse construction	[37]
Halton	Low-discrepancy sequence	Multidimensional	Modeling of groundwater monitoring networks.	[38]
		Multidimensional	Sensor placement in wireless networks.	[39]
Faure	Digital low-discrepancy sequence	Multidimensional	Proposal of a hybrid number generator.	[40]
		Multidimensional	Calculating eigenvalues in linear homogeneous integral equations.	[41]
Sobol	Digital low-discrepancy sequence	Multidimensional	Improving energy efficiency in stochastic computing circuits.	[42]

		Multidimensional	Generating numbers in linear time in Monte Carlo simulation.	[43]
Kronecker	Irrational-rotation sequence	Multidimensional	Generating numbers in the simulation of the differential dynamic logic (dL) algorithm.	[44]
		Multidimensional	Generating numbers in the simulation of oscillations that may occur due to obstacles encountered by vehicles on roads.	[45]
Hammersley	Finite low-discrepancy point set	Multidimensional	Proposing a multi-objective optimization method.	[46]

3.2.1. Pseudo-Random Initialization

Pseudo-random initialization generates each component of an initial candidate independently from a uniform distribution:

$$u_{i,j} \sim \mathcal{U}(0,1) \tag{S1}$$

where $i = 1, \dots, N$ denotes the candidate index and $j = 1, \dots, D$ denotes the problem dimension. The resulting unit-space samples are mapped to the search domain according to

$$x_{i,j} = l_j + u_{i,j}(u_j - l_j) \tag{S2}$$

where l_j and u_j are the lower and upper bounds of the $j - th$ decision variable.

Pseudo-random initialization is computationally simple and provides independent experimental replications. However, finite pseudo-random samples do not guarantee uniform multidimensional coverage and may contain clustered or sparsely represented regions. In this study, pseudo-random initialization is retained as the reference strategy against which structured and low-discrepancy methods are evaluated.

3.2.2. Latin Hypercube Sampling

Latin Hypercube Sampling is a stratified space-filling method in which the interval $[0,1)$ is divided into N equally sized strata for each dimension. One value is sampled from every stratum, and the resulting values are independently permuted across dimensions. A sample in the $j.$ dimension can be expressed as

$$u_{i,j} = \frac{\pi_j(i) - 1 + \xi_{i,j}}{N} \tag{S3}$$

where π_j is a random permutation of $\{1, \dots, N\}$, and $\xi_{i,j} \sim \mathcal{U}(0,1)$.

This construction ensures that every marginal interval contains one sample, thereby reducing one-dimensional clustering compared with unconstrained pseudo-random initialization. However, LHS does not necessarily minimize multidimensional discrepancy and should not be classified as a quasi-random sequence. It is included in this study as a stratified space-filling comparison strategy [20,23].

3.2.3. Van der Corput Sequence

The Van der Corput sequence is a one-dimensional low-discrepancy sequence based on the radical-inverse transformation [35,47–49]. Let the non-negative integer n be represented in base p, where p≥2, as follows:

$$n = (a_m \dots a_1 a_0)_p = \sum_{i=0}^{m} a_i p^i \qquad \text{(S4)}$$

Here, the coefficients a_i are in the range $0 \leq a_i < p$, and m is one less than the number of digits in the base p conversion. After the integer n is converted to base p, it is inverted and converted to decimal format. The resulting $\phi_p(n)$ value is expressed by Equation (S5):

$$X_n = \phi_p(n) = (0.a_0 a_1 \dots a_m)_p = \sum_{i=0}^{m} \frac{a_i}{p^{i+1}} \qquad \text{(S5)}$$

Because the canonical Van der Corput sequence is one-dimensional, it cannot be used directly as a conventional multidimensional point set. In the present study, a randomized coordinate-wise extension is employed, in which different index progressions are used across coordinates and the resulting design is randomized through a Cranley–Patterson shift. Therefore, the experimental variant is referred to as the randomized coordinate-wise Van der Corput extension.

The Van der Corput construction provides favorable one-dimensional distribution properties and constitutes the fundamental radical-inverse mechanism underlying several multidimensional low-discrepancy sequences [37].

3.2.4. Halton Sequence

The Halton sequence extends the radical-inverse construction of Van der Corput to multidimensional spaces by assigning a different prime base to each coordinate [35,47,50–55]. The i. point of a D-dimensional Halton sequence is expressed as

$$X_i = (\phi_{p_1}(i), \dots, \phi_{p_D}(i)) \qquad \text{(S6)}$$

Here, D represents the problem dimension, i the number of elements in the sequence, and p the number of consecutive prime numbers equal to the problem dimension.

Halton sequences generally provide effective space-filling behavior in low- and moderate-dimensional problems. However, correlations among coordinates associated with increasingly large prime bases may reduce their distribution quality as dimensionality increases. Scrambling procedures are therefore commonly used to reduce deterministic correlation patterns and support independent experimental replications [38, 39].

3.2.5. Faure Sequence

The Faure sequence is a multidimensional digital low-discrepancy construction that uses a common prime base p, typically selected such that $p \geq D$, across all dimensions [35,47,50]. Unlike the Halton sequence, which assigns a different prime base to each coordinate, the Faure construction applies coordinate transformations within a common prime base. The combination value c_j^i and the calculation of each dimension are performed with the following equations, respectively:

$$c_j^i = \binom{i}{j} mod(p) = \frac{i!}{j!\,(i-j)!} mod(p) \qquad \text{(S7)}$$

$$a_i^k(n) = \sum_{j=0}^{m} c_j^i . a_i^{k-1}(n) \;\; mod(p) \qquad \text{(S8)}$$

$$x_n^k = \phi_p^k(n) = \sum_{i=0}^{m} \frac{a_i^k(n)}{p^{i+1}} \quad 2 \leq k \leq s \qquad \text{(S9)}$$

Faure sequences were developed to provide a structured multidimensional digital construction and may reduce some of the coordinate-correlation limitations associated with high-dimensional Halton sequences. Their practical optimization performance, however, remains dependent on the problem structure and the implementation of the digital transformations [40, 41].

3.2.6. Sobol Sequence

A Sobol sequence is generated from binary functions of bits of the form $x_n = (x_{n,1}, \ldots, x_{n,d})$ and length ω. Each dimension of the Sobol sequence requires a different primitive polynomial. The general representation of this polynomial is as follows:

$$p_j(x) = x^q + a_1 x^{q-1} + \cdots + a_{q-1} x + 1 \tag{S10}$$

Next, a q-term recurrence relation is defined on the integers $M_{i,j}$:

$$M_{i,j} = 2a_1 M_{i-1,j} \oplus 2^2 a_2 M_{i-2,j} \oplus \ldots \oplus 2^{q-1} a_{q-1} M_{i-q+1,j} \oplus 2^q a_q M_{i-q,j} \oplus M_{i-q} \tag{S11}$$

Finally, the j. dimension of the n. term of the Sobol sequence is obtained with Equation (S12):

$$x_{n,j} = b_1 v_{1,j} \oplus b_2 v_{2,j} \oplus \ldots \oplus v_{\omega,j} \tag{S12}$$

Here, the $\oplus$ operator represents the XOR (Exclusive-OR) operation [35, 47, 50, 51]. Since only base-2 (p=2) numbers are generated when creating the Sobol sequence, they can be generated efficiently through bitwise operations and generally exhibit favorable discrepancy properties in multidimensional spaces. Nevertheless, their distribution quality depends on the selected direction numbers, scrambling procedure, sample size, and dimensionality. Therefore, Sobol initialization is evaluated empirically rather than being assumed to be superior in advance. It has shown superior performance in stochastic computation circuits [43] and linear-time simulations [42].

3.2.7. Kronecker Sequence

The Kronecker sequence is based on irrational numbers and their fractional parts. The fractional part of a number x is denoted by $\{x\} = x - \lfloor x \rfloor$. There is a certain upper bound for the discrepancy of this infinite sequence $(\{n\alpha\})_{n \leq 0}$ [50]:

$$D_n \leq C \frac{1 + \log n}{n} \tag{S13}$$

This sequence, which is effective in solving continuous (non-discrete) problems, has been used in trajectory plotting in medical robot-surgeon interaction simulations [44] and in vehicle-road interactive virtual reality simulations [45].

3.2.8. Hammersley Sequence

The Hammersley construction is a finite multidimensional low-discrepancy point set defined for a predetermined number of samples N. A D-dimensional Hammersley point can be represented as

$$x_i = \left(i/N, \phi_{p_1}(i), \ldots, \phi_{p_{n-1}}(i)\right), i = 1,2, \ldots, N \tag{S14}$$

Unlike Halton and Sobol sequences, which can be extended by generating additional terms, the Hammersley point set is explicitly constructed according to the required sample size N. This property may provide favorable finite-sample coverage but requires the population size to be specified before the point set is generated [46].

The initialization strategies described above exhibit different mathematical and geometric properties. Pseudo-random initialization provides stochastic independence, Latin Hypercube Sampling ensures marginal stratification, and the considered low-discrepancy constructions aim to improve multidimensional space coverage through different deterministic mechanisms. However, favorable geometric distribution does not necessarily guarantee superior optimization performance because the initial population subsequently interacts with the Mating, Branching, and Vaccinating operators and with the execution architecture of SGuA. Therefore, no initialization strategy is selected theoretically in advance. All eight strategies are evaluated independently on both SGuA1 and SGuA2 under identical population sizes, seeds, search bounds, and function-evaluation budgets. The strongest initialization candidates are determined through the Stage 1 experiments and are subsequently retained for hybrid screening.

Section S3. Chaotic-Map Definitions and Applications

Supplementary Table S2. Representative applications of the chaotic maps considered in the study.

Chaotic Map	Year	Problem	Ref.
Logistic Map	2019	Proposing a hybrid number generator for generating highly complex numbers.	[63]
	2020	Proposal of a modified logistic map for obtaining higher Lyapunov bases.	[64]
Circle Map	2016	Developing a good interleaver for Orthogonal Frequency Division Multiplexing.	[65]
	2017	Proposing a secure encryption algorithm for transmitting audio.	[66]
Gauss Map	2019	Proposing a chaotic particle swarm optimization method based on Gaussian maps for the classification of Parkinson's patients.	[67]
	2018	Proposing a new encryption algorithm for color images.	[68]
Sine Map	2019	Proposing a modified new sine map to increase complexity in image encryption and mixing operations.	[69]
	2019	A novel sine-based method is proposed to enable complex distributions of S-boxes (Substitution boxes).	[70]
Tent Map	2019	Improving the efficiency of the Harris Hawk optimization algorithm.	[71]
	2020	Increasing the complexity of the algorithm used to encrypt images.	[72]

In the map-specific definitions presented in this section, X_n denotes the generic scalar state of the corresponding chaotic map at iteration n. When a chaotic map is incorporated into HCQ-SGuA as an operator controller, the generated map state is represented by z_k in the main algorithmic formulation, where k denotes the internal controller-step index. The generated values are supplied as normalized control inputs to the Mating, Branching, and Vaccinating operators. Candidate solutions produced under these control inputs are subsequently evaluated using the objective function $f(x)$, the constraints $g_r(x)$, and the common selection score $S(x)$. Thus, X_n and z_k describe the generic chaotic-map state and its algorithmic controller representation, respectively, whereas x denotes the optimization decision vector. In the equations below, the right-hand-side expression defines the nonlinear state-update function of the corresponding chaotic map, whereas each complete equation constitutes the recurrence relation used to generate the map-state sequence.

3.3.1. Logistic Map

The mathematical model of the logistic map, one of the most fundamental chaotic maps in the literature, proposed in 1976 to model the nonlinear dynamics of biological populations, is presented in the following equation:

$$X_{n+1} = aX_n(1 - X_n), \ \ a = 4 \tag{S15}$$

Here, X_n represents the chaotic value in the $n-$th iteration (in the range $X_0 \in (0,1)$). It is the most applicable chaotic map due to its dimensional and simple structure. Although initially developed for population modeling, it is frequently preferred in optimization and cryptography today. To overcome the disadvantages of the limited range and low complexity of its standard form, many improved versions exist in the literature. For example, Luo et al. proposed a two-stage hybrid number generator integrated with a two-dimensional Baker map to increase the complexity of the Logistic map and developed a high-security image encryption algorithm [63].

Similarly, Moysis et al. presented a modified Logistic map with high Lyapunov exponents that successfully passed statistical tests (NIST) by manipulating the decimal parts of the map [64].

3.3.2. Circle Map

The Circle map proposed by Zheng is a one-dimensional map based on nonlinear oscillator dynamics and is expressed in Equation (S16):

$$X_{n+1} = X_n + 0.20 - (1.20/2\pi)\sin(2\pi X_n)\,mod(1) \tag{S16}$$

Although it has similar applications to the logistic map, it exhibits higher complexity due to its trigonometric component. A very small variation in the initial condition allows the generation of completely independent and unpredictable number sequences. Nair et al. have utilized the dispersion effect of the Circle map, which offers high randomness, to disperse burst errors in wireless data transmission in OFDM (Orthogonal Frequency Division Multiplexing) communication systems [65]. Kordov et al., on the other hand, proposed a cryptographically resistant encryption algorithm by subjecting the sequences obtained from two different Circle maps to an XOR operation to ensure the transmission security of voice data [66].

3.3.3. Gauss Map

A Gauss map is a piecewise chaotic function defined by Peitgen et al. and is often preferred in performance testing and safety applications in the literature:

$$X_{n+1} = \begin{cases} 0, & X_n = 0 \\ 1/X_n\,mod(1), & 0 < X_n < 1 \end{cases} \tag{S17}$$

Here the modulo operation is defined as in Equation (S18):

$$1/X_n\,mod(1) = \frac{1}{X_n} - \left\lfloor \frac{1}{X_n} \right\rfloor \tag{S18}$$

Gauss maps are primarily recommended for encryption algorithms due to the multitude of parameters they contain and their unpredictability. Koyuncu proposed a chaotic Particle Swarm Optimization (PSO) based on Gaussian maps for the classification of Parkinson's patients from voice recordings; he showed that it surpassed classical methods in evaluation criteria such as Accuracy, AUC and F-measure [67]. Sahay et al. also obtained more successful results than other chaotic maps in pixel scrambling operations using Gaussian maps in the encryption of color images [68].

3.3.4. Sine Map

The Sine map is a simple one-dimensional nonlinear state-update map defined over the unit interval. In the present study, it is expressed as

$$X_{n+1} = \sin(\pi X_n), \qquad 0 < X_n < 1 \tag{S19}$$

Its single-peak structure resembles the general form of the Logistic map, although the two maps employ different nonlinear state-update functions. The Sine map has been investigated in cryptographic sequence generation, image scrambling, and substitution-box construction [69,70].

3.3.5. Tent Map

The Tent map is a piecewise-linear state-update map whose graphical representation resembles a tent. The symmetric Tent map used in the present study is defined as:

$$X_{n+1} = \begin{cases} 2X_n, & 0 < X_n < 0.5 \\ 2(1 - X_n), & 0.5 < X_n < 1 \end{cases} \tag{S20}$$

The map expands the current state differently on either side of the midpoint and maps the resulting value back into the unit interval. Tent-map dynamics have been incorporated into several swarm-intelligence and cryptographic frameworks [28,30,71,72].

Section S4. Initialization and Chaotic-Sequence Validity Diagnostics

3.2.9. Initialization-Quality Diagnostics

Although visual inspection provides an intuitive comparison of population initialization strategies, it is not sufficient for quantitatively evaluating their distribution quality, particularly in multidimensional search spaces [35]. Therefore, the initial populations generated by the eight strategies considered in this study were also examined using complementary geometric diagnostics. Let

$$U = [u_1, u_2, \ldots, u_N]^T \in [0,1)^{NxD} \quad \text{(S21)}$$

denote an initial population generated in the normalized unit search space, where N is the population size and D is the problem dimension. The following diagnostic measures were calculated for each generated population.

Centered Discrepancy: Centered discrepancy measures the deviation of a finite point set from an ideally uniform distribution over the unit hypercube. Lower discrepancy values indicate that the generated population covers the search space more uniformly and contains fewer overrepresented or insufficiently represented regions. In this study, centered discrepancy was used as the principal quantitative indicator of multidimensional distribution uniformity.

Minimum Pairwise Distance: The minimum Euclidean distance between any two distinct population members was calculated as

$$d_{min} = \min_{1 \le i < j \le N} \left\| u_i - u_j \right\|_2 \quad \text{(S22)}$$

A larger d_{min} value indicates that the closest two candidate solutions are more widely separated, thereby reducing excessive clustering and duplicated regional coverage. However, this metric reflects only the closest pair and should therefore be interpreted together with discrepancy and correlation measures.

Dimensional Rank: The rank of the centered population matrix was calculated to determine whether the generated samples span the available dimensional structure. Let

$$\widetilde{U} = U - 1\mu^T \quad \text{(S23)}$$

where

$$\mu = \frac{1}{N}\sum_{i=1}^{N} u_i \quad \text{(S24)}$$

is the coordinate-wise mean vector. The dimensional rank is then expressed as

$$r_U = rank(\widetilde{U}) \quad \text{(S25)}$$

A rank approaching $min(N-1, D)$ indicates that the population is not restricted to a lower-dimensional subspace. A reduced rank may reveal redundant coordinate patterns or insufficient dimensional diversity.

Coordinate Correlation: Pairwise correlations between the dimensions of the generated population were examined to identify undesirable dependencies among coordinates. For the set of absolute pairwise correlation values

$$\mathcal{R} = \{ |p_{jk}| : 1 \le j < k \le D \} \quad \text{(S26)}$$

the mean and maximum absolute coordinate correlations were calculated as

$$\bar{p} = \frac{1}{|\mathcal{R}|}\sum_{p \in \mathcal{R}} p \quad \text{(S27)}$$

and

$$p_{max} = \max_{p \in \mathcal{R}} p \quad \text{(S28)}$$

Lower values of $\bar{p}$ and p_{max} indicate weaker linear dependence among dimensions and a more independent representation of the search space. High maximum correlation values may reveal structured alignments that are not immediately apparent from marginal distributions.

Taken together, lower centered discrepancy, larger minimum pairwise distance, full dimensional rank, and lower coordinate correlations generally indicate a more representative initial population. Nevertheless, these geometric characteristics do not necessarily guarantee superior optimization performance because the generated

population subsequently interacts with the Mating, Branching, and Vaccinating operators and with the SGuA1 or SGuA2 execution architecture. Therefore, the diagnostic measures are used to explain the spatial behavior of the initialization strategies rather than to determine the winning strategy independently. Final initialization selection is based on the optimization results obtained under identical population sizes, seeds, search bounds, and function-evaluation budgets.

3.3.6. Chaotic-Sequence Validity Diagnostics

The visual appearance of a chaotic trajectory alone is not sufficient to determine whether the generated sequence is numerically valid or suitable for controlling an optimization operator. Therefore, the five chaotic maps considered in this study were subjected to a common diagnostic procedure before their integration into SGuA.

For each map, the initial state was generated in the interval $x_0 \sim U(0.05, 0.95)$. An initial burn-in period was then applied to reduce the direct influence of the initial transient behavior. After the burn-in stage, a sufficiently long sequence was generated and examined using the following diagnostic measures:

- arithmetic mean;
- standard deviation;
- observed minimum and maximum values;
- lag-one autocorrelation;
- normalized permutation entropy;
- finite-time Lyapunov estimate; and
- the proportion of finite values remaining within the required interval.

The lag-one autocorrelation was used to examine short-term linear dependence between consecutive chaotic states. Permutation entropy was employed to characterize the temporal complexity of the generated trajectory [88], whereas the finite-time Lyapunov estimate was used as an indicator of sensitivity to nearby initial states [89].

A chaotic sequence was accepted as numerically valid only when all generated values were finite, remained within the required numerical interval after normalization, and exhibited nondegenerate variation. Maps producing collapsed, constant, non-finite, or numerically unstable trajectories were not considered valid operator controllers.

These diagnostics were used only to verify the numerical behavior of the chaotic sequences. They were not used independently to select the winning map because favorable sequence characteristics do not necessarily guarantee superior optimization performance.

Section S5. Exact Mathematical Models of the Engineering Problems

S5.1. Tension/Compression Spring

Let $x = (d, D_c, N_c)$, where d is wire diameter, D_c is mean coil diameter, and N_c is the number of active coils.

$$min\, f(x) = (N_c + 2)D_c d^2$$
$$g_1(x) = 1 - {D_c}^3 N_c/(71785 d^4) \leq 0$$
$$g_2(x) = [4{D_c}^2 - dD_c]/[12566(D_c d^3 - d^4)] + 1/(5108 d^2) - 1 \leq 0$$
$$g_3(x) = 1 - 140.45d/({D_c}^2 N_c) \leq 0$$
$$g_4(x) = (d + D_c)/1.5 - 1 \leq 0$$
$$0.05 \leq d \leq 2.0, 0.25 \leq D_c \leq 1.3, 2 \leq N_c \leq 15.$$

S5.2. Pressure Vessel

Let $x = (T_s, T_h, R, L)$, where T_s and T_h are shell and head thicknesses, R is inner radius, and L is cylindrical-section length.

$$min\, f(x) = 0.6224 T_s RL + 1.7781 T_h R^2 + 3.1661 {T_s}^2 L + 19.84 {T_s}^2 R$$
$$g_1(x) = 0.0193R - T_s \leq 0;$$
$$g_2(x) = 0.00954R - T_h \leq 0$$
$$g_3(x) = 1296000 - \pi R^2 L - \left(\frac{4}{3}\right)\pi R^3 \leq 0;$$

$$g_4(x) = L - 240 \leq 0$$

$0.0625 \leq T_s, T_h \leq 6.1875, 10 \leq R \leq 200, 10 \leq L \leq 240$; T_s and T_h are repaired to integer multiples of 0.0625.

S5.3. Welded Beam

Let $x = (h, l, t, b)$, where h is weld thickness, l is weld length, t is beam height, and b is beam thickness.

$$min\, f(x) = 1.10471h^2l + 0.04811tb(14 + l)$$
$$g_1(x) = \tau(x) - 13600 \leq 0;$$
$$g_2(x) = \sigma(x) - 30000 \leq 0;$$
$$g_3(x) = h - b \leq 0$$
$$g_4(x) = 0.10471h^2 + 0.04811tb(14 + l) - 5 \leq 0;$$
$$g_5(x) = 0.125 - h \leq 0$$
$$g_6(x) = \delta(x) - 0.25 \leq 0;$$
$$g_7(x) = 6000 - Pc(x) \leq 0$$
$$\tau = \sqrt{\left[\tau'^2 + \frac{2\tau'\tau''l}{2R} + \tau''^2\right]}, \tau' = \frac{6000}{\sqrt{2hl}},$$
$$\tau'' = \frac{MR}{J}, M = 6000\left(14 + \frac{l}{2}\right), R = \sqrt{\left[\frac{l^2}{4} + \frac{(h+t)^2}{4}\right]},$$
$$J = 2\sqrt{2hl\left[\frac{l^2}{12} + \frac{(h+t)^2}{4}\right]}$$
$$\sigma = \frac{504000}{t^2b}, \delta = \frac{2.1952}{t^3b},$$
$$Pc = 64746.022(1 - 0.0282346t)t\, b^3$$
$$0.1 \leq h, b \leq 2.0\ and\ 0.1 \leq l, t \leq 10.0.$$
